# A comprehensive review on the chemical constituents, sesquiterpenoid biosynthesis and biological activities of *Sarcandra glabra*

**DOI:** 10.1007/s13659-023-00418-8

**Published:** 2023-11-27

**Authors:** Jin-Ning Chu, Premanand Krishnan, Kuan-Hon Lim

**Affiliations:** 1 School of Pharmacy, University of Nottingham Malaysia, Jalan Broga, 43500 Semenyih, Selangor Malaysia; 2 Foundation in Science, University of Nottingham Malaysia, Jalan Broga, 43500 Semenyih, Selangor Malaysia

**Keywords:** *Sarcandra glabra*, CaoShanHu, Traditional Chinese medicine, Lindenane-type sesquiterpenoids, Biosynthetic pathway, Biological activities

## Abstract

*Sarcandra glabra* (Thunb.) Nakai is a perennial evergreen herb categorised within the *Sarcandra Gardner* genus under the Chloranthaceae family. Indigenous to tropical and subtropical regions of East Asia and India, this species is extensively distributed across China, particularly in the southern regions (Sichuan, Yunnan, and Jiangxi). In addition to its high ornamental value, *S. glabra* has a rich history of use in traditional Chinese medicine, evident through its empirical prescriptions for various ailments like pneumonia, dysentery, fractures, bruises, numbness, amenorrhea, rheumatism, and other diseases. Besides, modern pharmacological studies have revealed various biological activities, such as antitumour, anti-bacterial, anti-viral anti-inflammatory and immunomodulatory effects. The diverse chemical constituents of *S. glabra* have fascinated natural product researchers since the 1900s. To date, over 400 compounds including terpenoids, coumarins, lignans, flavonoids, sterols, anthraquinones, organic acids, and organic esters have been isolated and characterised, some featuring unprecedented structures. This review comprehensively examines the current understanding of *S. glabra*’s phytochemistry and pharmacology, with emphasis on the chemistry and biosynthesis of its unique chemotaxonomic marker, the lindenane-type sesquiterpenoids.

**Graphical Abstract:**

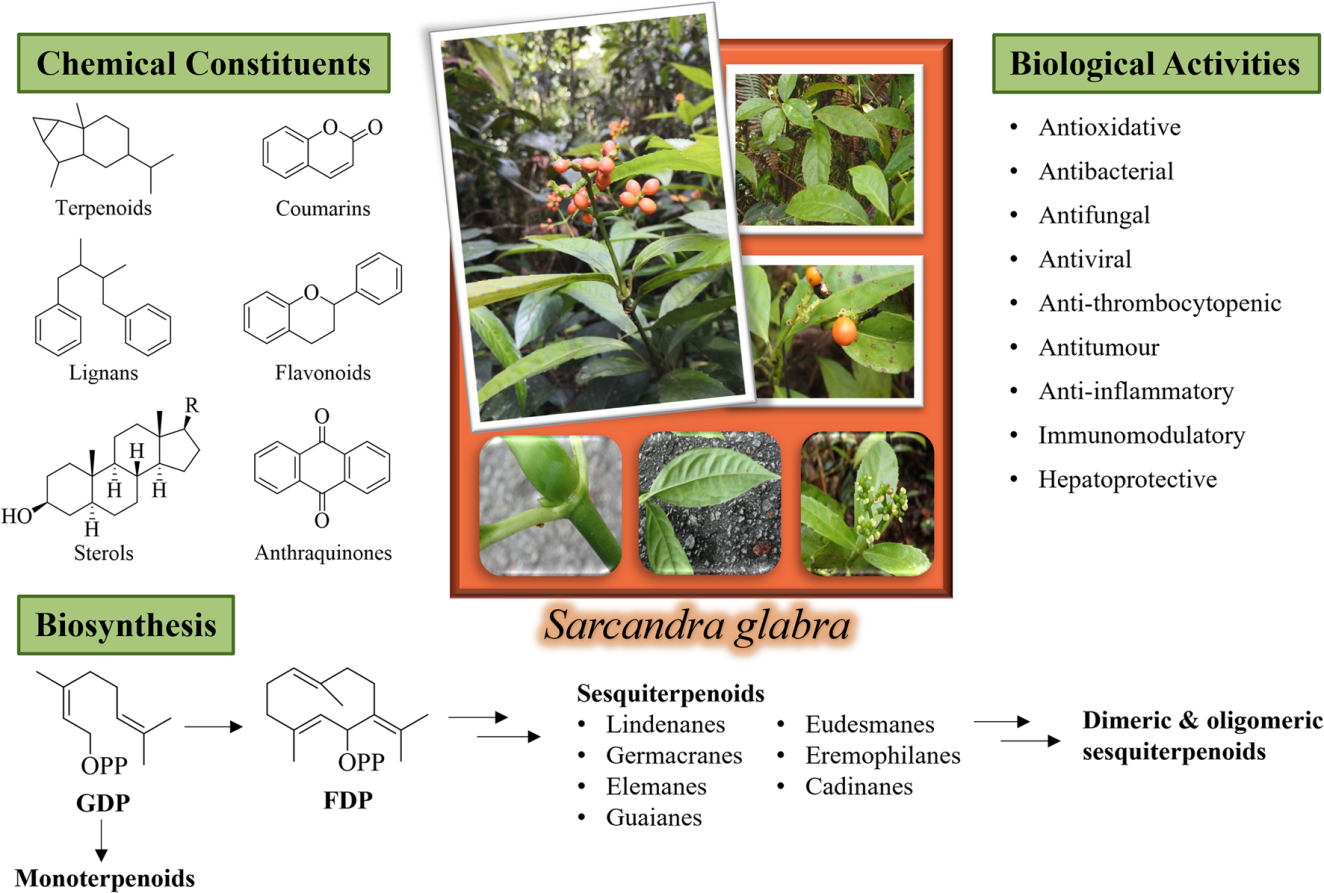

## Introduction

*Sarcandra* (*Sarcandra* Gardner) is a genus under the family of Chloranthaceae. The genus name, *Sarcandra*, is integrated from ‘Sarkos’ and ‘andrus’, which means fleshy anthers in Greek, while the epithet ‘glabra’ translates to ‘hairless’ from Latin [1]. *Sarcandra glabra* (Thunb.) Nakai (syn. *Chloranthus glaber* (Thunb.) Makino) or *Sarcandra glabra* in short, represents an extensively researched species of the genus *Sarcandra*. The plant is an evergreen shrub that grows up to 2 m tall and has glossy green leaves with a distinctive aroma. The plant is occasionally planted for ornamental purposes, otherwise used to prepare medicinal tea.

Also referred to as 草珊瑚 (CaoShanHu) in Chinese*, S. glabra* is valued in Traditional Chinese Medicine for its immunomodulatory [2], anti-inflammatory [3], and anti-tumour properties [4], and used to treat a variety of health conditions, including arthritis, bronchitis, and cancer [5]. The distribution of the subshrub ranges from temperate East Asia to Southeast China, specifically in provinces such as Anhui, Fujian, Guangdong, Guangxi, Guizhou, Hainan, Zhejiang, and Sichuan [1]. Ecologically, *S. glabra* thrives in geographic locations that are approximately 2000 m above sea level and can be found plentiful in forests, thickets, valleys, ravines, trail sides, grasslands, and swamps [1].

Within the *S. glabra* species, two subspecies are officially accepted, namely *Sarcandra glabra* subsp. *glabra* and *Sarcandra glabra* subsp. *brachystachys* [6]*. S. glabra* subsp. *glabra*, known as 原亚种 (YuanYaZhong) in Chinese, is a subspecies indigenous to continental East Asia, including North and Central China, Korea, Japan and the Ryukyu Islands. *Sarcandra chloranthoides* Gardner is treated as a synonym of this taxon and its distribution is centred in India and Sri Lanka [1].

The second subspecies, *S. glabra* subsp. *brachystachys* (Blume) has a widespread distribution in Northeast India, Northern Vietnam, Southern China and throughout the Malesian region. *Sarcandra hainanensis* (Pei) Swamy & Bailey (海南草珊) and *Chloranthus brachystachys* Blume are two synonyms of this taxon and are sometimes used interchangeably [1]. This subspecies differs from subsp. *glabra* by the length of its anther being almost as equal as the whole male structure, while in subsp. *glabra* the anther is much shorter and the non-antheriferous part is well-developed [7, 8]. Anatomically, the ventral vein in subsp. *hainanensis* is single, as opposed to the paired strands in subsp. *glabra* [9]. *Sarcandra glabra* var. *melanocarpa* (Ridl.) Verdc. or *Chloranthus brachystachys* var. *melanocarpus* Ridl. is a lesser-known variation of *S. glabra* subsp. *brachystachys*. It is an endemic plant found in the montane rainforests of North Sumatra and Malesia and is characterised by its unique black fruits [6].

Since the mid-twentieth century, the diverse chemical constituents of *S. glabra* have piqued the scientific curiosity of various researchers. The observed diversity in the chemical constituents of *S. glabra* may be attributed to its proliferative nature and location-specific environmental factors influencing the plant’s growth and metabolism. To date, studies have reported nearly 400 compounds from this species, including terpenoids, coumarins, lignans, flavonoids, sterols, anthraquinones, organic acids, and organic esters, many of which have been found to possess interesting structures and/or significant pharmacological activities. These findings underscore the potential of *S. glabra* as a vast resource for drug discovery and its development.

While several published review papers have covered the phytochemistry of *S. glabra* [5, 10, 11], there remains a gap in individual and comprehensive reviews that specifically address its chemical constituents and the associated biogenetic pathways. As more recent work surfaced, this review aims to provide a categorical progress update (up to August 2023) on the isolation and structural elucidation of chemical constituents of *S. glabra,* along with the proposed biosynthetic pathways of specific dimeric and oligomeric sesquiterpenoids, and the biogenetic relationship among these terpenoid skeletons. Furthermore, this review covers an overview of the pharmacological and clinical exploration of crude extracts, medicinal preparations, and the bioactive compounds of *S. glabra*.

## Chemical constituents

### Isolation of terpenoids from *S. glabra*

As the most widely occurring and extensively studied family in natural products, terpenoids are generally distinguished by the number of isoprene (C_5_) units constituting their carbon skeleton [12]. The term terpenoids refers to modified terpenes that have been chemically altered through oxidation or rearrangement, and is occasionally used interchangeably with terpenes [12]. More than 200 terpenoids have been reported from *S. glabra,* including triterpenoids (C_30_), diterpenoids (C_20_), sesquiterpenoids (C_15_), monoterpenoids (C_10_), as well as meroterpenoids.

#### Sesquiterpenoids

Among the isolates from *S. glabra*, sesquiterpenoids constitute the largest proportion with more than 180 members. The structures of sesquiterpenoids from *S. glabra* are rather diverse and accompanied by complex stereochemistry. The structures of these sesquiterpenoids can be divided into eight main skeletal types, namely, eudesmane, lindenane, germacrane, eremophilane, aromadendrane, elemane, guaiane, and cadinene. Herein, the structures of all sesquiterpenoids isolated up to August 2023 are presented; however, only those that were first isolated from *S. glabra* are highlighted. 

##### Eudesmane and eudesmane dimers

**Fig. 1:**
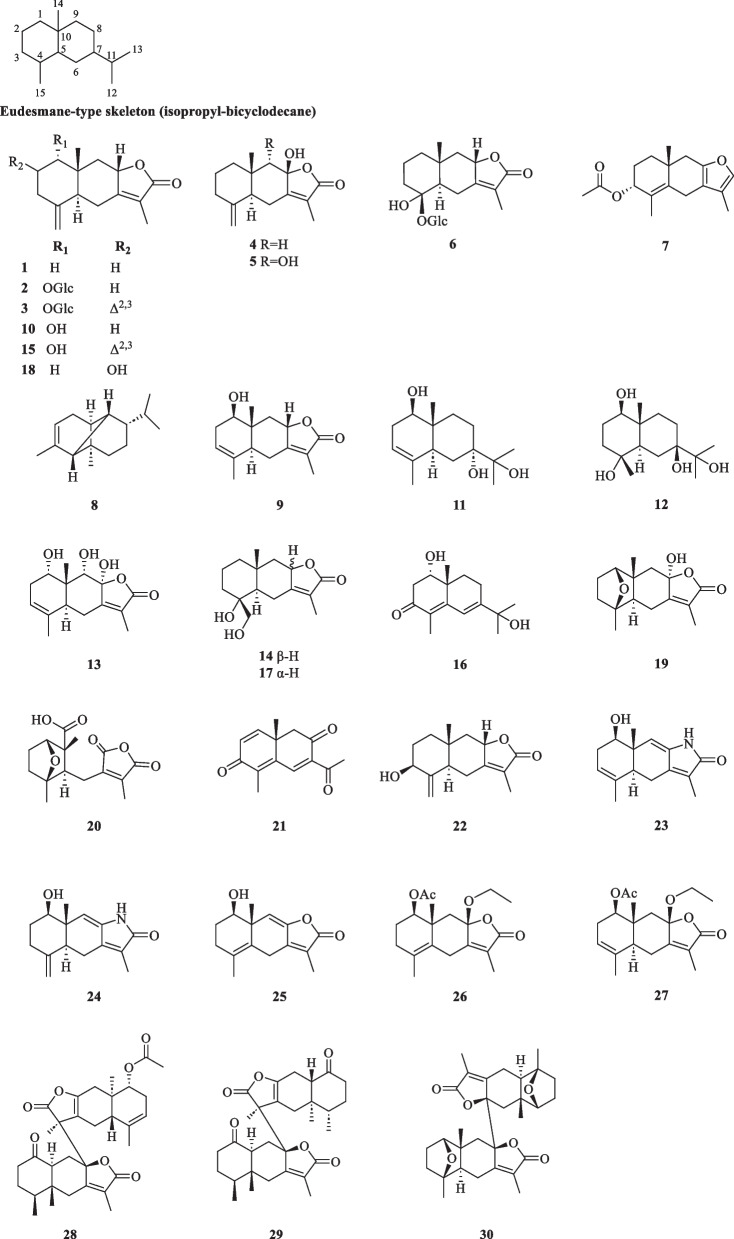
Eudesmane-type sesquiterpenoids and eudesmane-type dimers (**1**–**30**)

Known as selinanes in the early literature, the basic skeleton of eudesmane features an isopropyl-bicyclodecane with four asymmetric centres at C-4, C-5, C-7, and C-10 [13]. The formation of a lactone moiety involving C-7, C-8, C-11, and C-12 gives rise to eudesmane-type sesquiterpene lactones known as eudesmanolides, which account for the majority of the eudesmane-type sesquiterpenoids obtained from *S. glabra.* Currently, a total of 30 eudesmane compounds (**1**–**30**) have been reported (Table 1, Fig. 1), the majority of which were monomers isolated from the whole plants and leaves of *S. glabra* (**1**–**27**).

**Table 1:** Eudesmane-type sesquiterpenoids and dimers from *S. glabra*

Eudesmane-type sesquiterpenoids and dimers	Molecular formula	Source	Fraction	References
1. Atractylenolide II	C_15_H_20_O_2_	*C. glaber*, leaves	Et_2_O	[26]
2. Sarcaglaboside A	C_21_H_30_O_8_	*S. glabra*, whole plant (Dayu county, Jiangxi province)	EtOH	[14]
3. Sarcaglaboside B	C_21_H_28_O_8_			
4. 8β-Hydroxyeudesma-4(15),7(11)-dien-12,8-olide	C_15_H_20_O_5_	*S. glabra,* whole plant (Dayu county, Jiangxi province)	EtOH	[15]
5. 8β,9α-Dihydroxyeudesman-4(15),7(11)-dien-8α,12-olide	C_15_H_20_O_4_			
6. Sarcaglaboside H	C_21_H_32_O_9_	*S. glabra*, whole plant (Xiushui, Jiangxi province)	EtOH	[16]
7. 3α-Acetoxy-8,12-epoxyeudesma-4,7,11-triene	C_17_H_22_O_3_	*S. glabra*, leaves (Penang Hill, Penang)	Essential oil	[27]
8. α-Copaene	C_15_H_24_			
9. Sarcandralactone B / serralactone A	C_15_H_20_O_3_	*S. glabra*, whole plant (Hainan province)	EtOAc	[17]
10. Neolitacumone B	C_15_H_20_O_3_			
11. 3-Eudesmene-1*β*,7,11-triol	C_15_H_26_O_3_			
12. Glabranol B	C_15_H_28_O_4_	*S. glabra,* aerial part (Vinh Phuc province, Vietnam)	EtOAc	[19]
13. 1α,8α,9α-Trihydroxyeudesman-3(4),7(11)-dien-8β,12-olide	C_15_H_20_O_5_	*S. glabra*, whole plant	EtOH	[20]
14. Atractylenolide IV	C_15_H_23_O_4_	*S. glabra*, whole plant (Jiujiang, Jiangxi province)	EtOAc	[21]
15. Sarcandralactone E	C_15_H_18_O_3_	*S. glabra*, whole plant (Guilin, Guangxi province)	EtOAc	[22]
16. ‘Compound 3’ (3-Oxo-eudesma-4,6-diene-1α,11-diol)	C_15_H_22_O_3_	*S. glabra*, seeds (Ganzhou, Jiangxi province)	EtOAc	[23]
17. ‘Compound 4’ (4α,15-Dihydroxy-5α,8αH-eudesman-7(11)-en-8α,12-olide)	C_15_H_22_O_4_			
18. ‘Compound 5’ (2α-Hydroxy-eudesma-4(15),7(11) diene-8,12-olide)	C_15_H_20_O_3_			
19. Sarglanoid D	C_15_H_20_O_4_	*S. glabra*, whole plant (Ganzhou, Jiangxi province)	DCM	[24]
20. Sarglanoid E	C_15_H_18_O_6_			
21. Linderaggredin D	C_14_H_14_O_3_			
22. 3β-Hydroxyeudesma-4(15),7(11)-dien-8α,12-olide	C_15_H_20_O_3_			
23. Sarglanoid A	C_15_H_19_NO_2_	*S. glabra*, leaves (Guangxi province)	DCM, PE	[25]
24. Sarglanoid B	C_15_H_19_NO_2_			
25. Sarglanoid C	C_15_H_18_O_3_			
26. Sarglanoid D	C_19_H_26_O_5_			
27. Sarglanoid E	C_19_H_26_O_5_			
28. Sarglanoid A	C_32_H_40_O_7_	*S. glabra,* whole plant (Ganzhou, Jiangxi province)	DCM	[24]
29. Sarglanoid B	C_30_H_38_O_6_			
30. Sarglanoid C	C_30_H_38_O_6_

Sarcaglabosides A and B (**2** and** 3**) were the first examples of hepatoprotective compounds reported from the whole plants of *S. glabra* [14]*.* Zhu et al. [15] reported compound **5** as the 9α-hydroxy eudesmanolide derivative of **4**, while Hu et al. [16] reported compound **6** as a new eudesmanolide glycoside from the whole plant of *S. glabra*. Sancandralactone B (**9**) was reported as a new compound from *S. glabra* by He et al. [17] but it was originally characterised as serralactone A from *Chloranthus serratus* [18]*.* Glabranol B (**12**) is a new eudesmane-type sesquiterpenoid separated from the aerial parts of a Vietnamese *S. glabra* specimen [19]. Wang et al. [20] reported the trihydroxyeudesmanolide derivative **13**, while Hu et al. [21] reported the 4,15-glycol derivative **14** from the whole plant of *S. glabra*. Designated as the aglycone of sarcaglaboside B (**3**), sarcandralactone E (**15**) was obtained from the whole plant of *S. glabra* of Guangxi origin [22]*.* The presence of three eudesmane compounds (**16**–**18**) was detected in the seeds of *S. glabra* [23], with **16** obtained as a racemic mixture and **17** characterised as the C-8 epimer of **14**. The *S. glabra* (whole plant) collected from Jiangxi gave four eudesmane-type sesquiterpenoids (**19**–**22**) [24], among which compounds **19** and **20** represent eudesmanolides incorporating a rare 1,4-epoxy bridge. The same plant also yielded compounds **28** and **29**, which were the first heterodimer representatives isolated from a *Sarcandra* plant that incorporate eudesmane and eremophilane sesquiterpenoid halves, along with compound **30**, which is a symmetric homodimer featuring a different dimerisation pattern from the former dimeric compounds [24].

A chemical investigation of the leaves of *S. glabra* from Guangxi province led to the discovery of compounds **23**–**27**, with compounds **23** and **24** being unusual γ-lactam-containing eudesmane-type sesquiterpenoids [25]. Unfortunately, while compounds **28**–**30**, **19** and **20** were given the trivial names sarglanoids A-E, respectively, compounds **23**–**27** were coincidentally given the same names as both sets of compounds were published around the same time.

##### Lindenane and lindenane oligomers

Despite the limited distribution of lindenane-type sesquiterpenoids in natural sources, their presence is exceptionally prominent in *S. glabra*. The occurrences of lindenane-type sesquiterpenoids as oligomers, including homodimers, heterodimers, and trimers in *S. glabra* have gained much research interest due to their intriguing structures. Among them, sarcanolides, sarcandralactones, sarglabolides, sarglalactones, sarcaglabrins and sarcaglabosides could serve as the characteristic components and important chemotaxonomic markers of *S. glabra* due to their species-wide exclusivity [5].

In monomeric form, lindenane sesquiterpenoids possess a common skeleton comprising a unique linear 3/5/6 tricyclic ring. The system is embedded with a chiral carbon (C-10), an atypical *trans*-5/6 junction, and a sterically congested cyclopentane with an angular C-14 methyl group [28]. The structural variation of lindenane oligomers is attributed to the combination of monomeric units that make up the backbone, which could be assembled by different linkage patterns. Generally, most dimeric lindenanes from *S. glabra* were proposed to be constructed from *endo*-Diels–Alder reactions [29].

**Fig. 2:**
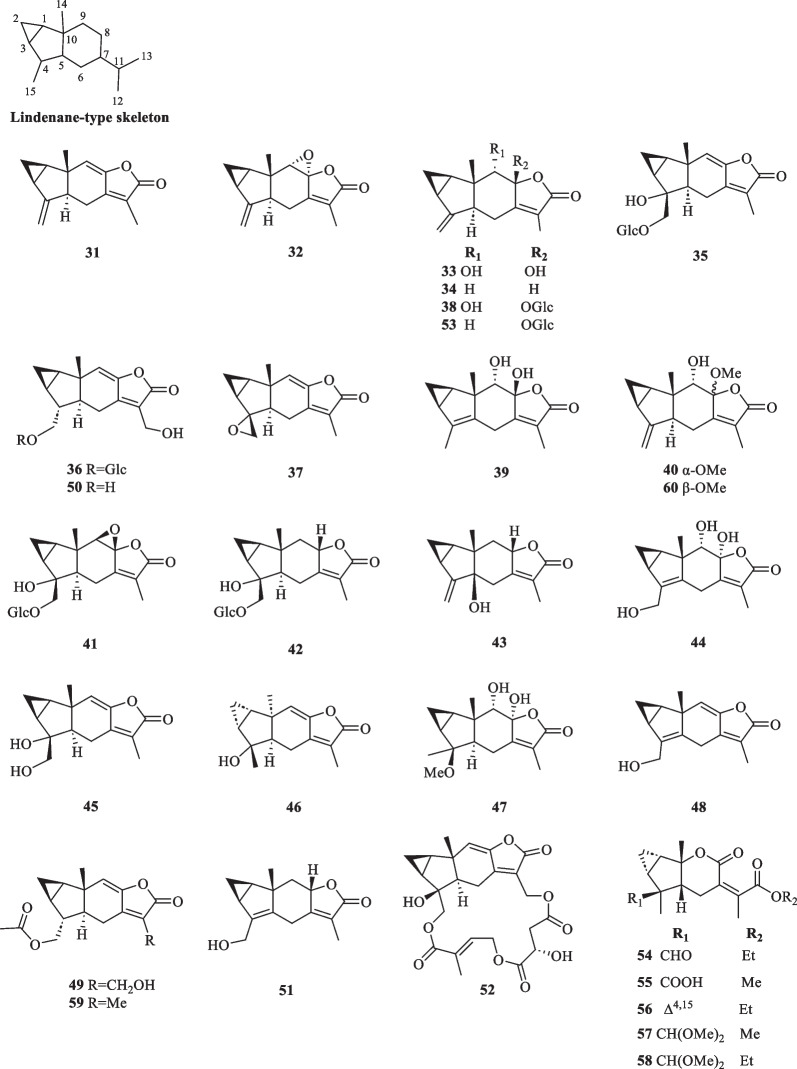
Lindenane-type sesquiterpenoids (**31**–**60**)

At present, 30 lindenane-type sesquiterpenoids (**31**–**60**) from *S. glabra* were reported, among which lindenane-type sesquiterpene lactones (lindenanolides) account for a significant proportion (Table 2, Fig. 2). The first lindenane-type sesquiterpenes (**31**–**32**) were isolated by Uchida et al. [30] from *Chloranthus glaber* (synonym of *S. glabra*). Subsequently, the presence of several known lindenane compounds (**33**–**39**) was reported from the same plant [14, 15, 26, 31, 32]. The whole plants of *S. glabra* from Xiushui, Jiangxi yielded two new lindenane glycosides, sarcaglabosides F and G (**41** and **42**), together with a known compound (**40**) [16]. In search for cytotoxic sesquiterpenoids from a Hainanese *S. glabra* plant, He et al. [17] reported sarcandralactone A (**43**), which is a C-5 hydroxylated analogue of shizukanolide A (**34**). The butanolic extract of *S. glabra* (whole plant) provided a new trihydoxylindenanolide, glabranol A (**44**) [19]. Compounds **47** and **48** were reported as new compounds from *S. glabra* by Ni et al. [22], and were named sarcandralactones C and D, respectively. A phytochemical investigation of the anti-inflammatory constituents from *S. glabra* led to the discovery of sarglabolide L (**52**), representing a rare lindenane derivative possessing an 18-membered macrocyclic triester, and shizukanolide 8-*O*-β-D glucopyranoside (**53**) [23]. From the dichloromethane and petroleum ether fractions of *S. glabra*, Chi et al. [33] reported the isolation of sarglalactones I-M (**54**–**58**), a group of unique 8,9-secolindenane derivatives featuring an opened lactone ring.

**Table 2:** Lindenane-type sesquiterpenoids from *S. glabra*

Lindenane-type sesquiterpenoids	Molecular formula	Source	Fraction	References
31. Chloranthalactone A / shizukanolide B	C_15_H_16_O_2_	*C. glaber,* roots	Et_2_O	[30]
32. Chloranthalactone B	C_15_H_15_O_3_			
33. Chloranthalactone E	C_15_H_18_O_4_	*C. glaber,* leaves	Et_2_O	[26]
34. Shizukanolide A	C_15_H_18_O_2_	*C. glaber,* roots	Ether-pentane	[31]
35. Chloranoside A / shizukanolide E 15-*O*-β-glucoside	C_21_H_28_O_9_	*C. glaber,* whole plant	Acetone	
36. Chloranoside B / shizukanolide F 15-*O*-β-glucoside	C_21_H_28_O_9_			
37. Chloranthalactone G	C_15_H_16_O_3_	*S. glabra*, whole plant	DCM	[32]
38. Chloranthalactone E 8-*O*-β-D-glucopyranoside	C_21_H_28_O_9_	*S. glabra*, whole plant (Dayu county, Jiangxi province)	EtOH	[14]
39. 8β,9α-Dihydroxylindan-4(5),7(11)-dien-8α,12-olide	C_15_H_18_O_4_	*S. glabra*, whole plant	EtOH, Acetone	[15]
40. 9-Hydroxy-heterogorgiolide	C_15_H_20_O_4_	*S. glabra*, whole plant (Xiushui, Jiangxi province)	Acetone, EtOH	[16]
41. Sarcaglaboside F	C_21_H_28_O_10_			
42. Sarcaglaboside G	C_21_H_30_O_9_			
43. Sarcandralactone A	C_15_H_18_O_3_	*S. glabra*, whole plant (Hainan province)	EtOAc	[17]
44. Glabranol A	C_15_H_18_O_5_	*S. glabra*, whole plant (Vinh Phuc province, Vietnam)	BuOH	[19]
45. Shizukanolide E	C_15_H_18_O_4_	*S. glabra*, whole plant	EtOH, Acetone	[34]
46. 4α-Hydroxy-5α-H-lindan-8(9)-en-8,12-olide	C_15_H_18_O_3_	*S. glabra*, whole plant	EtOH	[35]
47. Sarcandralactone C	C_16_H_22_O_5_	*S. glabra*, whole plant (Guilin, Guangxi province)	EtOAc	[22]
48. Sarcandralactone D	C_15_H_16_O_3_			
49. Shizukanolide H	C_17_H_20_O_5_			
50. Shizukanolide F	C_15_H_18_O_4_	*S. glabra*, whole plant	Acetone	[21]
51. Chlorajapolide C	C_15_H_18_O_3_	*S. glabra*, whole plant	EtOAc	[36]
52. Sarglabolide L	C_24_H_26_O_10_	*S. glabra*, seeds (Ganzhou, Jiangxi province)	EtOAc	[23]
53. Shizukanolide 8-*O*-β-D glucopyranoside	C_21_H_28_O_8_			
54. Sarglalactone I	C_17_H_22_O_5_	*S. glabra*, leaves (Guangxi province)	Polar DCM, PE	[33]
55. Sarglalactone J	C_16_H_20_O_6_			
56. Sarglalactone K	C_16_H_20_O_4_			
57. Sarglalactone L	C_18_H_26_O_6_			
58. Sarglalactone M	C_19_H_28_O_6_			
59. Chloranthalactone C	C_17_H_20_O_4_	*S. glabra*, aerial part (Guangxi province)	MeOH, EtOAc	[4]
60. Chlorajapolide F	C_16_H_20_O_4_

**Fig. 3:**
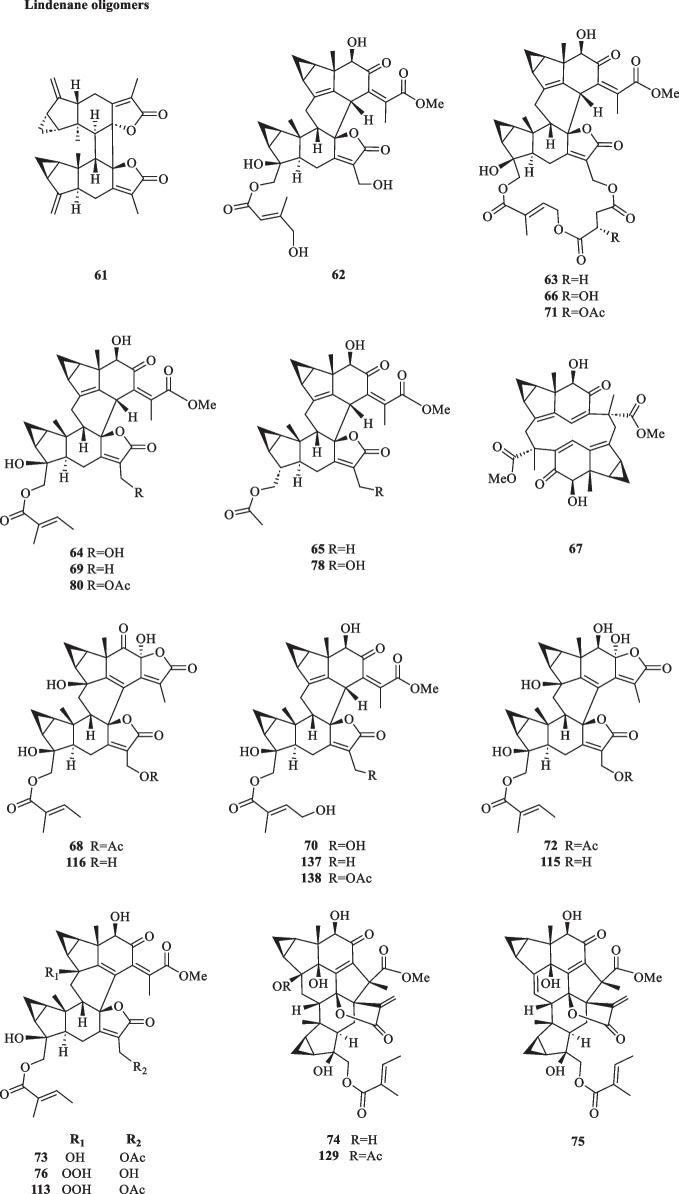

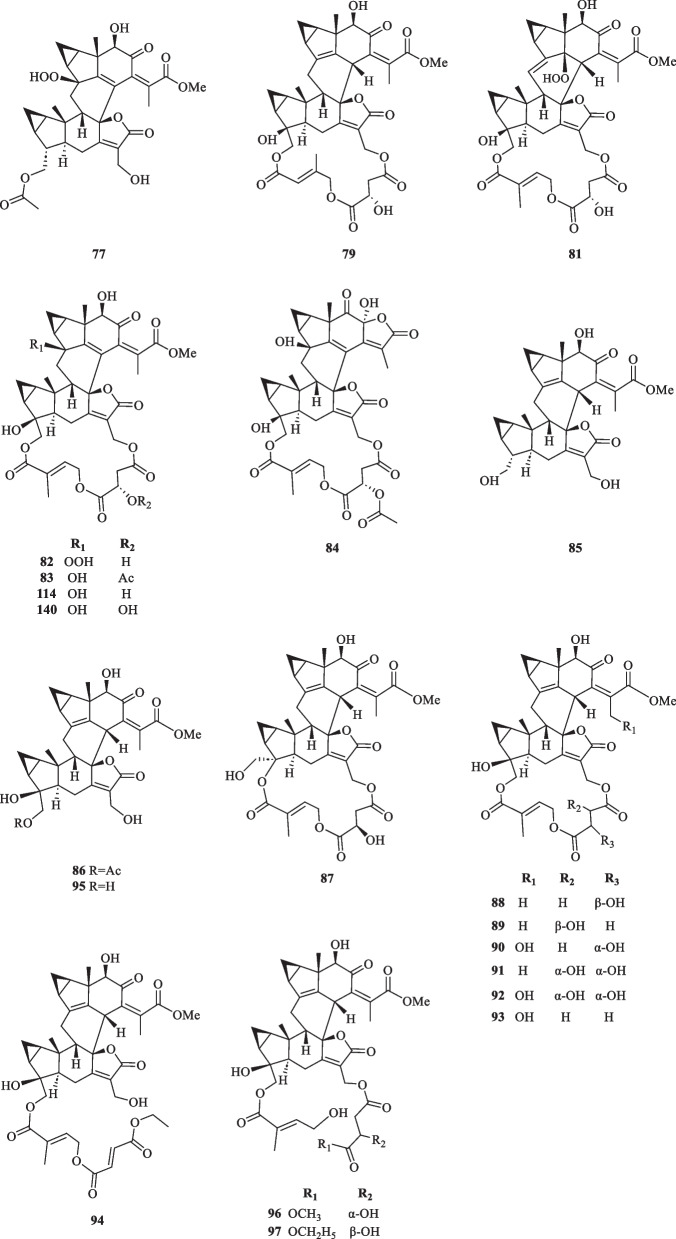

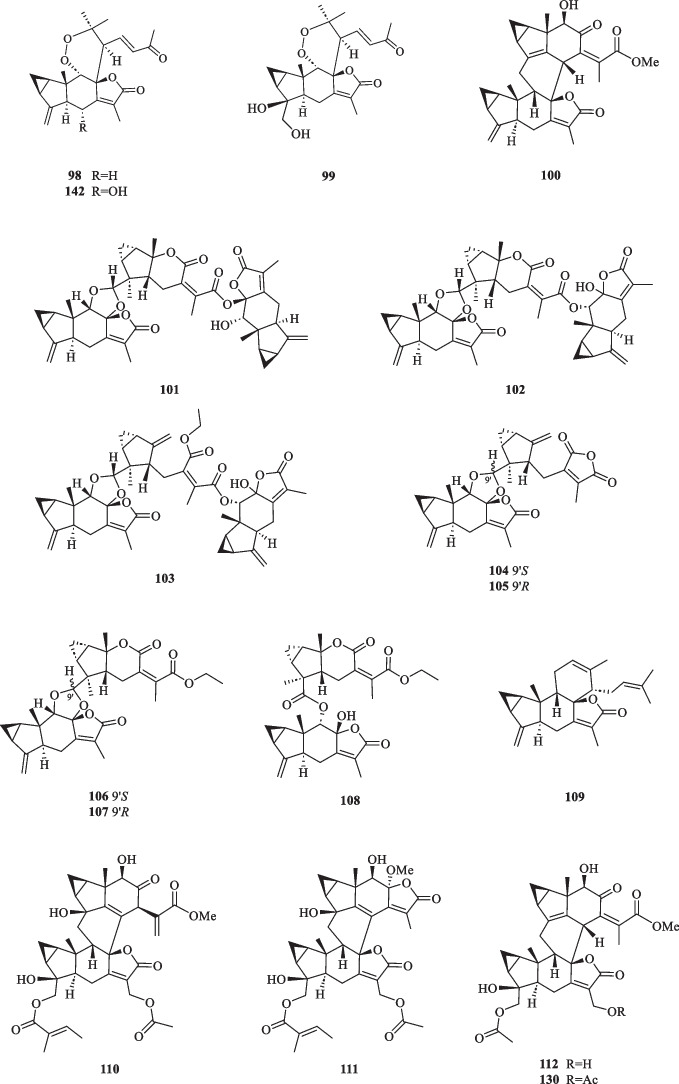

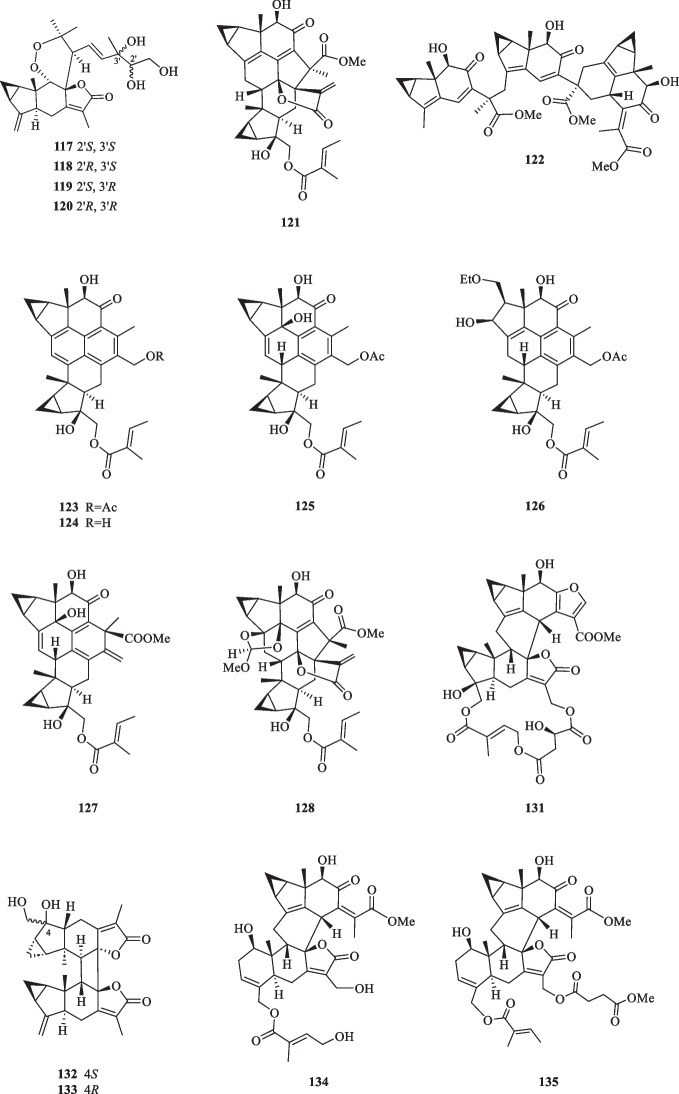

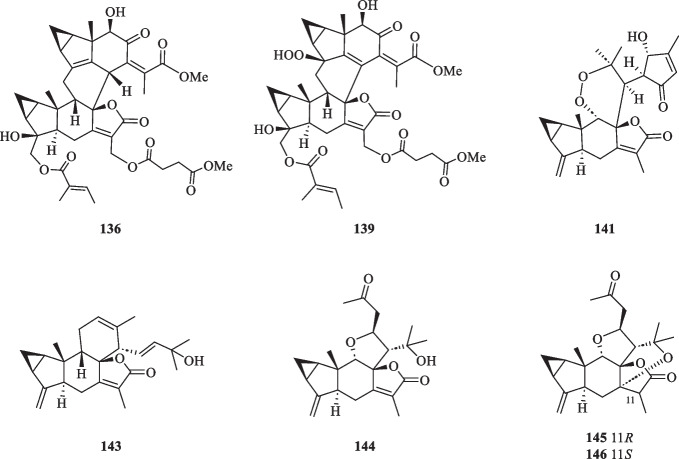
Lindenane oligomers (**61**–**146**)

Lindenane-type sesquiterpenoid oligomers and their derivatives are recognised as the characteristic taxonomic symbol of *S. glabra*. Despite having complex structures and functionalities, 86 lindenane-type sesquiterpenoid oligomers (**61**–**146**) were successfully characterised from *S. glabra* (Table 3, Fig. 3).

**Table 3:** Lindenane oligomers from *S. glabra*

Lindenane oligomers	Molecular formula	Source	Fraction	References
61. Chloranthalactone A photodimer / chloranthalactone F	C_30_H_32_O_4_	*C. glaber,* leaves	Et_2_O	[26]
62. Shizukaol I	C_36_H_42_O_11_	*S. glabra*, whole plant	EtOH	[46]
63. Shizukaol B/ henriol C	C_40_H_44_O_13_	*S. glabra,* whole plant, seeds	EtOAc	[17, 37]
64. Shizukaol C	C_36_H_42_O_10_			
65. Shizukaol E	C_33_H_38_O_8_			
66. Shizukaol G	C_40_H_44_O_14_			
67. Cycloshizukaol A	C_32_H_36_O_8_			
68. Chlorahololide F	C_37_H_40_O_12_			
69. Sarcandrolide A (13′-deoxyshizukaol C)	C_36_H_42_O_9_			
70. Sarcandrolide B	C_36_H_42_O_11_			
71. Sarcandrolide C (2′′′-*O*-acetylshizukaol G)	C_42_H_46_O_15_			
72. Sarcandrolide D	C_37_H_42_O_12_			
73. Sarcandrolide E	C_38_H_44_O_12_			
74. Sarcanolide A	C_36_H_42_O_11_	*S. hainanensis,* whole plant	EtOAc	[47]
75. Sarcanolide B	C_36_H_40_O_10_			
76. Chlorajaponilide E	C_36_H_42_O_12_	*S. glabra,* whole plant (Guilin, Guangxi province)	EtOAc	[22]
77. Spicachlorantin F	C_33_H_38_O_11_			
78. Shizukaol D	C_33_H_38_O_9_			
79. Shizukaol H	C_40_H_44_O_14_			
80. Henriol D / chlorahololide D	C_38_H_44_O_11_			
81. Sarcandrolide F	C_40_H_44_O_16_			
82. Sarcandrolide G	C_40_H_44_O_16_			
83. Sarcandrolide H	C_42_H_46_O_16_			
84. Sarcandrolide I	C_41_H_42_O_16_			
85. Sarcandrolide J	C_31_H_36_O_8_			
86. Shizukaol N	C_33_H_38_O_10_	*S, glabra,* seeds (Ganzhou, Jiangxi province)	EtOAc	[37]
87. Sarglabolide A	C_40_H_44_O_14_			
88. Sarglabolide B	C_40_H_44_O_14_			
89. Sarglabolide C	C_40_H_44_O_14_			
90. Sarglabolide D	C_40_H_44_O_15_			
91. Sarglabolide E	C_40_H_44_O_15_			
92. Sarglabolide F	C_40_H_44_O_16_			
93. Sarglabolide G	C_40_H_44_O_14_			
94. Sarglabolide H	C_42_H_48_O_14_			
95. Sarglabolide I	C_31_H_36_O_9_			
96. Sarglabolide J	C_41_H_48_O_15_			
97. Sarglabolide K	C_42_H_50_O_15_			
98. Sarglaperoxide A	C_23_H_28_O_5_	*S. glabra,* seeds	PE	[38]
99. Sarglaperoxide B	C_23_H_30_O_7_	*S. glabra,* seeds	EtOAc	
100. Shizukaol A	C_31_H_34_O_6_	*S. glabra,* roots		[48]
101. Sarglalactone A	C_45_H_50_O_11_	*S. glabra,* leaves (Guangxi province)	PE	[33]
102. Sarglalactone B	C_45_H_50_O_11_			
103. Sarglalactone C	C_47_H_54_O_11_			
104. Sarglalactone D	C_30_H_32_O_7_			
105. Sarglalactone E	C_30_H_32_O_7_			
106. Sarglalactone F	C_32_H_38_O_8_			
107. Sarglalactone G	C_32_H_38_O_8_			
108. Sarglalactone H	C_32_H_38_O_9_			
109. Sarcaglabrin A	C_25_H_32_O_2_	*S. glabra,* aerial part (Guangxi province)	MeOH, EtOAc	[4]
110. Sarcaglabrin B	C_38_H_44_O_12_			
111. Sarcaglabrin C	C_38_H_44_O_12_			
112. Multistalide B	C_33_H_38_O_10_			
113. Spicachlorantin E	C_38_H_44_O_13_			
114. Chloramultilide A	C_40_H_44_O_14_			
115. Chloramultilide D / henriol B	C_35_H_40_O_11_			
116. Chloramultiol D	C_35_H_38_O_11_			
117. Sarcaglarol A	C_25_H_34_O_7_	*S. glabra,* leaves (Guangxi province)	PE, DCM	[39]
118. Sarcaglarol B	C_25_H_34_O_7_			
119. Sarcaglarol C	C_25_H_34_O_7_			
120. Sarcaglarol D	C_25_H_34_O_7_			
121. Fortunilide K	C_36_H_40_O_9_	*S. glabra,* whole plant (Bozhou, Anhui province)	EtOAc	[49]
122. Trishizukaol A	C_48_H_54_O_12_	*S. glabra,* roots	DCM	[50]
123. Sarglaromatic A	C_35_H_38_O_7_	*S. glabra*, roots (Sanming, Fujian province)	DCM	[41]
124. Sarglaromatic B	C_33_H_36_O_6_			
125. Sarglaromatic C	C_35_H_40_O_8_			
126. Sarglaromatic D	C_37_H_46_O_9_			
127. Sarglaromatic E	C_35_H_40_O_8_			
128. Sarcanolide C	C_38_H_44_O_12_	*S. glabra*, roots (Yingjiang county, Yunnan province)	EtOAc	[42]
129. Sarcanolide D	C_38_H_44_O_12_			
130. Sarcanolide E	C_35_H_40_O_11_			
131. Sarglafuran A	C_40_H_42_O_14_	*S. glabra*, leaves (Liuzhou, Guangxi province)	DCM	[43]
132. Sarglalactone N	C_30_H_34_O_6_			
133. Sarglalactone O	C_30_H_34_O_6_			
134. 15′-*O*-(4-Hydroxytigloyl)fortunoid C	C_36_H_42_O_11_	*S. glabra* subsp*.* *brachystachys,* whole plant (Hainan)	EtOAc	[44]
135. 13′-*O*-Methyl succinyl-15′-*O*-tigloylfortunoid C	C_41_H_48_O_13_			
136. 13′-*O*-Methylsuccinylshizukaol C	C_41_H_48_O_13_			
137. 4′′-Hydroxysarcandrolide A	C_36_H_42_O_10_			
138. 13′-*O*-Acetylsarcandrolide B	C_38_H_44_O_12_			
139. 13′-*O*-Methylsuccinylchlorajaponilide E	C_41_H_48_O_15_			
140. (7′′*S*)-7′′-Hydroxychloramultilide A	C_40_H_44_O_15_			
141. Sarcaglarone A	C_25_H_30_O_6_	*S. glabra*, seeds (Sanming, Fujian province)	PE	[40]
142. 6α-Hydroxysarglaperoxide A	C_23_H_28_O_6_			
143. 7′-Oxyisosarcaglabrin A	C_25_H_32_O_3_			
144. Sarglaoxolane A	C_23_H_30_O_5_	*S. glabra*, leaves (Guangxi province)	PE	[45]
145. Sarglaoxolane B	C_23_H_30_O_5_			
146. Sarglaoxolane C	C_23_H_30_O_5_

Although most of the dimeric lindenane sesquiterpenoids from *S. glabra* were presumed to be biosynthesised via Diels–Alder, several compounds displayed variations in the linkage between two constitutional units. Chloranthalactone F (**61**), later renamed as chloranthalactone A photodimer, is a representative dimeric sesquiterpenoid formed by a [2 + 2] cycloaddition [26]. Being the only [6 + 6] lindenane cycloadduct isolated from *S. glabra*, cycloshizukaol A (**67**) has an interesting C-2-symmetrical structure that incorporates a cyclododecatetraene ring [17]. He et al. [17] isolated a group of structurally related lindenane dimers known as sarcandrolides A-E (**69**–**73**) from the whole plant of *S. glabra.* The same group also isolated two new dimers, sarcanolides A and B (**74** and **75**), featuring an unprecedented nonacyclic scaffold from *S. hainanensis* (subspecies of *S. glabra*).

Ni et al. [22] reported five new dimeric lindenanes, sarandrolides F-J (**81**–**85**), together with five known compounds (**76**–**80**) in the course of cytotoxicity screening of *S. glabra* sesquiterpenoids. Sarcandrolide F (**81**) represents the first example of a lindenane-type dimer with a hydroperoxy group at C-5 [22]. Wang et al. [37] investigated the seeds of *S. glabra,* which led to the isolation of 11 new lindenane dimers, sarglabolide A-K (**87**–**97**). Among the isolates, compound **87** has a notable 17-membered macrocyclic ester ring that differs from the usual 18-membered system featured in other lindenane dimers. The continued endeavour of Wang’s group [38] resulted in the discovery of two uncommon heterodimers, sarglaperoxides A and B (**98** and **99**), featuring a lindenane and a normonoterpene unit assembled via a 1,2-dioxane ring, from the seeds of *S. glabra*. With the guidance of MS/MS molecular networking, Wang et al. [39] reported the occurrence of four additional isomeric heterodimers, sarcaglarols A-D (**117**–**120**), whose skeletal structures resemble those of **98** and **99**, from the leaves of *S. glabra.* Another structurally related heterodimer sarcaglarone A (**141**) was isolated by Sun et al. [40] from the seeds of *S. glabra*, which incorporates a distinctive lactone in the monoterpene fragment.

An investigation by Chi et al. [33] on *S. glabra* from Guangxi afforded a series of unprecedented 8,9-secolindenane-type sesquiterpenoid oligomers, including three trimers, sarglalactones A-C (**101**–**103**), and five dimers, sarglalactones D-H (**104**–**108**). *S. glabra* specimens from the Guangxi region were also found to contain a rare lindenane-monoterpene heterodimer sarcaglabrin A (**109**), two new lindenane dimers sarcaglabrins B and C (**110**–**111**), and five known compounds (**112**–**116**) [4]. Sun et al. [41] reported the isolation of five norlindenane dimers, sarglaromatics A-E (**123**–**127**), bearing a naphthalene or a dihydronaphthalene core from the roots of *S. glabra,* whereas Xiao et al. [42] reported the addition of three new sarcanolides (**128**–**130**) from the roots of *S. glabra.* Compared to their congeners (**74** and **75**), sarcanolide C (**128**) bears a supplemental orthoformate ring at C-4 and C-5, whereas sarcanolide D (**129**) was elucidated as the 4-*O*-acetyl derivative of sarcanolide A (**74**). From the leaves of *S. glabra*, Wang et al. [43] encountered an unprecedented [4 + 2]-type lindenane dimer, sarglafuran A (**131**), which represents the first dimer to contain a furan moiety in its lindenane monomer unit instead of an α,β-unsaturated lactone or an opened-ring lactone. Wang’s group [43] also reported the isolation of sarglalactones N and O (**132** and **133**), a pair of C-4 epimers that also possess a [2 + 2]-type dimeric skeleton and were identified as dihydroxy derivatives of **61**.

Zhou et al. [44] revealed the presence of seven new dimeric lindenanes (**135**–**140**) with significant antimalarial activities and a known compound (**134**) from the roots of *S. glabra* subsp. *brachystachys*. 15′-*O*-4-Hydroxytigloylfortunoid C (**134**) and 13′-*O*-methyl succinyl-15′-*O*-tigloylfortunoid C (**135**) share a common heterodimeric core comprising a lindenane and a eudesmane unit, whereas 4″-hydroxysarcandrolide A (**137**) and 13′-*O*-acetylsarcandrolide B (**138**) were identified as structural analogues of **70**. 6α-Hydroxysarglaperoxide A (**142**) and 7′-oxyisosarcaglabrin A (**143**) are two new heterodimers isolated from the seeds of *S. glabra,* and were determined as the derivatives of **98** and **109**, respectively [40]. Guided by a single-node-based molecular networking approach, Cui et al. [45] discovered the first tetrahydrofuran-linked-lindenane-normonoterpene heterodimer sarglaoxolane A (**144**) from the leaves of *S. glabra*, together with a pair of pseudonatural epimers sarglaoxolanes B and C (**145**–**146**), which were spontaneously formed from **144**.

##### Germacranes

**Fig. 4:**
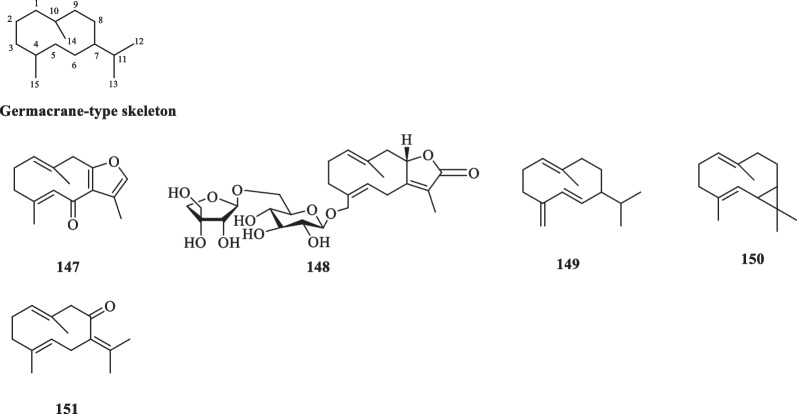
Germacrane-type sesquiterpenoids (**147**–**151**)

Germacranes are an elemental class of sesquiterpenoids containing a characteristic cyclodecane ring formed from the ring closure of C-1 and C-10 of farnesane [51]. The carbon skeleton features an isopropyl group at C-7 and two methyl groups at C-4 and C-10 [52]. Five germacrane-type sesquiterpenoids (**147**–**151**) have been isolated from the essential oils and ethanolic extract of *S. glabra* (Table 4, Fig. 4), with sarcaglaboside E (**148**) being identified as a new glucoside derivative [14]*.*

**Table 4:** Germacrane-type sesquiterpenoids from* S. glabra*

Germacrane-type sesquiterpenoids	Molecular formula	Source	Fraction	References
147. Furanodienone	C_15_H_18_O_2_	*S. glabra*, whole plant	EtOH, essential oil	[27, 53]
148. Sarcaglaboside E	C_26_H_38_O_12_	*S. glabra*, whole plant (Dayu county, Jiangxi province)	EtOH	[14]
149. Germacrene D	C_15_H_24_	*S. glabra*, leaves (Penang Hill, Penang)	Essential oil	[27]
150. Bicyclogermacrene	C_15_H_24_			
151. Germacrone	C_15_H_22_O

##### Eremophilanes

**Fig. 5:**
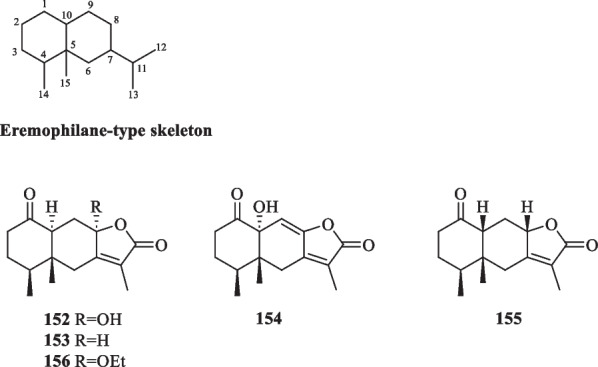
Eremophilane-type sesquiterpenoids (**152**–**156**)

Eremophilanes belong to a family of sesquiterpenoids featuring a bicyclodecane skeleton similar to that of eudesmane-type sesquiterpenoids. The carbon skeleton of eremophilane is naturally derived from the rearrangement of eudesmane derivatives, exemplified by the migration of the methyl group from position C-10 to C-5 [51, 54]. The number of eremophilane-type sesquiterpenoids isolated from *S. glabra* amounts to five so far (Table 5, Fig. 5), four of which were first isolated from other plants, i.e., **152**–**155**. Sarglanoid F (**156**), the 8-ethoxy derivative of **153**, is the only eremophilane that was first reported from *S. glabra* [25]*.*

**Table 5:** Eremophilane-type sesquiterpenoids from *S. glabra*

Eremophilane-type sesquiterpenoids	Molecular formula	Source	Fraction	References
152. (−)-Istanbulin A	C_15_H_20_O_4_	*S. glabra*, leaves	EtOAc, EtOH	[15, 55]
153. Istanbulin B	C_15_H_20_O_3_	*S. glabra*, whole plant	EtOAc	[36, 56]
154. 10α-Hydroxy-1-oxoeremophila-7(11),8(9)-dien-8,12-olide	C_15_H_18_O_4_	*S. glabra,* whole plant (Ganzhou, Jiangxi province)	DCM	[24]
155. 1-Oxo-10β(H)-eremophila-7(11)-en-8α,12-olide	C_15_H_20_O_3_			
156. Sarglanoid F	C_17_H_24_O_4_	*S. glabra,* leaves (Guangxi province)	DCM, PE	[25]

##### Aromadendranes

**Fig. 6:**
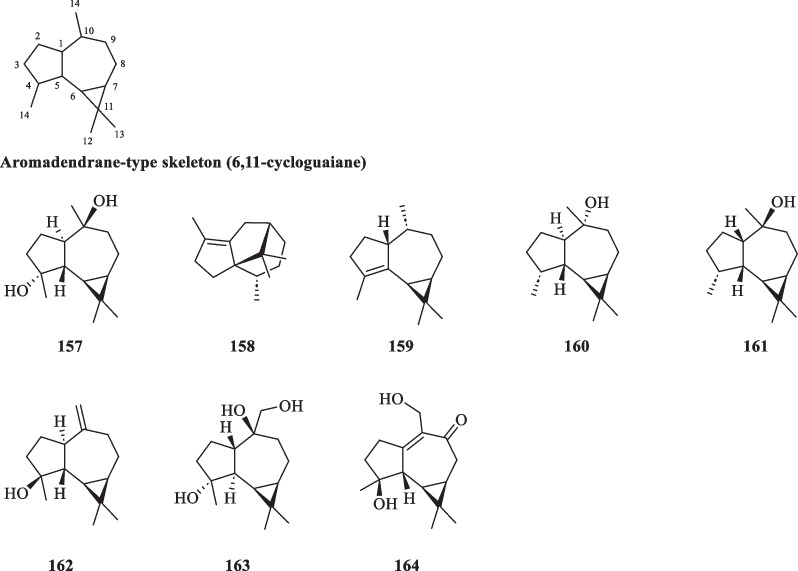
Aromadendrane-type sesquiterpenoids (**157**–**164**)

The scaffold of aromadendranes usually incorporates a *gem*-dimethylcyclopropane ring that is fused to a hydroazulene skeleton [57]. The skeleton bears a strong resemblance to that of guaiane-type sesquiterpenoids, differing only in having a cyclopropane ring formed by the bond linking C-6 and C-11 in aromadendranes. Hence, aromadendrane-type sesquiterpenoids are also known as 6,11-cycloguaianes [51]. At present, eight aromadendrane-type sesquiterpenoids (**157**–**164**) from *S. glabra* were documented in the literature (Table 6, Fig. 6). Sarglanoid G (**164**) was reported for the first time from the roots of *S. glabra* [58].

**Table 6:** Aromadendrane-type sesquiterpenoids from *S. glabra*

Aromadendrane-type sesquiterpenoids	Molecular formula	Source	Fraction	References
157. (-)-4β,7α-Dihydromadendrane / aromadendrane-4-β,10-α-diol	C_15_H_26_O_2_	*C. glaber,* leaves	Et_2_O	[26]
158. Cyperene	C_15_H_24_	*S. glabra*, leaves (Penang Hill, Penang)	Essential oil	[27]
159. α-Gurjunene	C_15_H_24_			
160. Globulol	C_15_H_26_O			
161. Viridiflorol	C_15_H_26_O			
162. Spathulenol	C_15_H_24_O	*S. glabra*, whole plant	Essential oil, EtOAc	[17, 27]
163. Pipelol A	C_15_H_26_O_3_	*S. glabra*	EtOH	[59]
164. Sarglanoid G	C_15_H_22_O_3_	*S. glabra,* roots	-	[58]

##### Elemanes

**Fig. 7:**
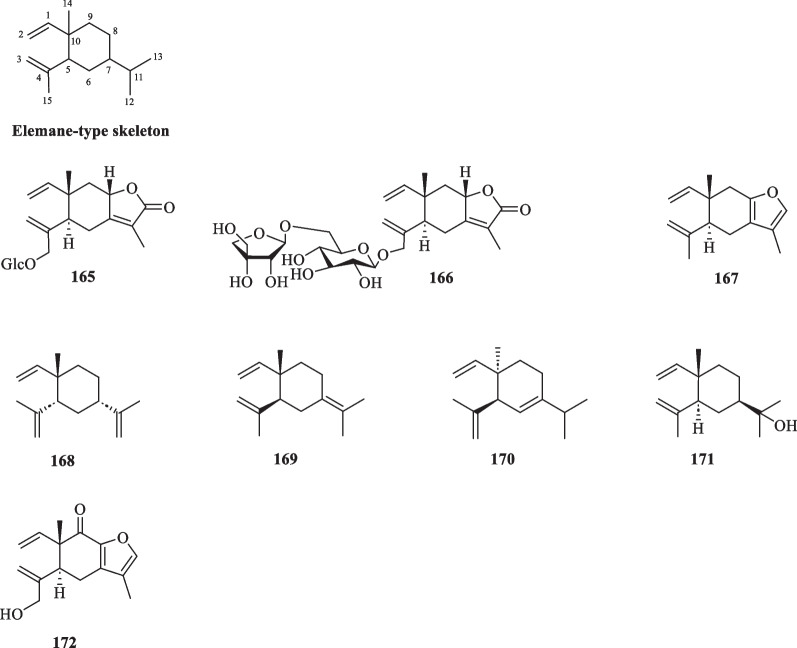
Elemane-type sesquiterpenoids (**165**–**172**)

Elemanes constitute a small group of sesquiterpenoids in *S. glabra* and are olefinic compounds with cyclohexane as their core [60]. A total of eight sesquiterpenes of this class (**165**–**172**) have been reported from *S. glabra* (Table 7, Fig. 7), including two new elemanolide aglycons, sarcaglaboside C and D (**165** and **166**)[14], five known compounds (**167**–**171**), and a new furan-bearing elemane-type sesquiterpenoid sarglanoid H (**172**) [58].

**Table 7:** Elemane-type sesquiterpenoids from *S. glabra*

Elemane-type sesquiterpenoids	Molecular formula	Source	Fraction	References
165. Sarcaglaboside C	C_21_H_30_O_8_	*S. glabra*, whole plant (Dayu county, Jiangxi province)	EtOH	[14]
166. Sarcaglaboside D	C_26_H_38_O_12_			
167. Curzerene	C_15_H_20_O	*S. glabra,* leaves (Penang Hill, Penang)	Essential oil	[27, 61]
168. β-Elemene	C_15_H_24_			
169. γ-Elemene	C_15_H_24_			
170. δ-Elemene	C_15_H_24_			
171. Elemol	C_15_H_26_O			
172. Sarglanoid H	C_15_H_18_O_3_	*S. glabra,* roots	-	[58]

##### Guaiane, cadinane, and other sesquiterpenoids

**Fig. 8:**
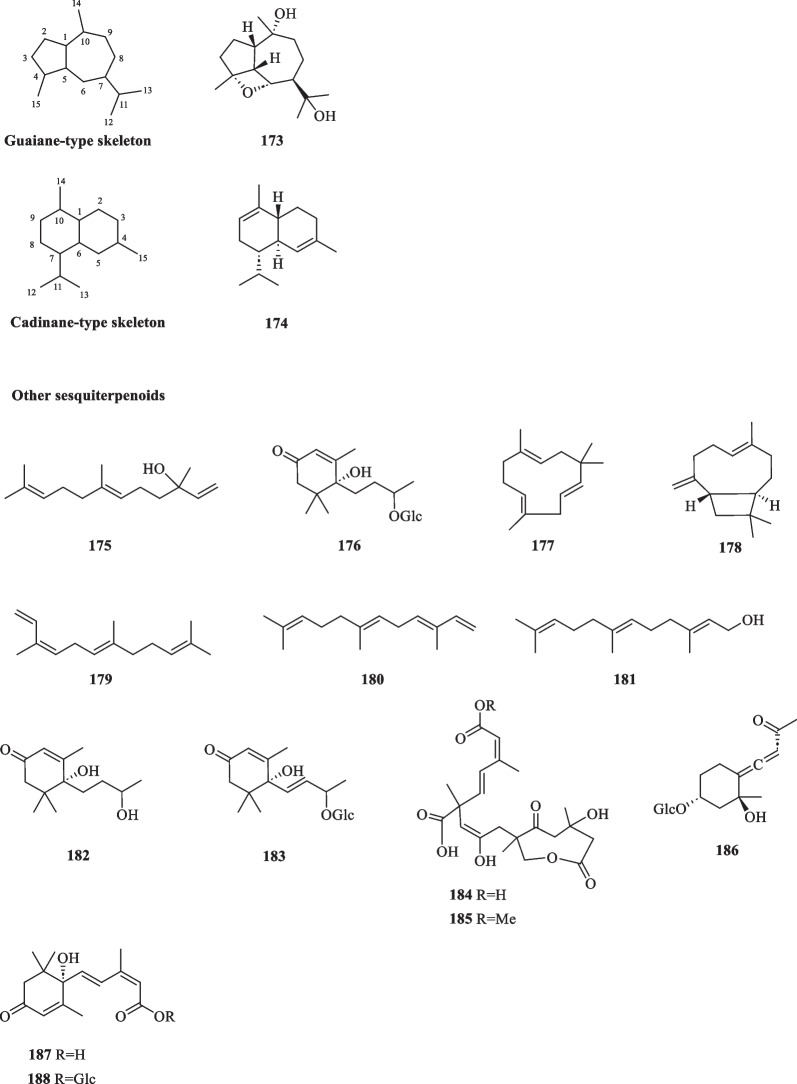
Guaiane-type sesquiterpenoid (**173**), cadinane-type sesquiterpenoid (**174**), and other sesquiterpenoids (**175**–**188**)

Compound **173** is the only guaiane-type sesquiterpenoid isolated from the whole plant of *S. glabra* featuring a distinctive epoxy bridge at C-4 and C-6, whereas compound **174**, a component of cade oil [62], is the only cadinane-type sesquiterpenoid isolated from the essential oil of *S. glabra* (Table 8, Fig. 8).

**Table 8:** Guaiane, cadinane, and other sesquiterpenoids from *S. glabra*

Chemical constituents	Molecular formula	Source	Fraction	References
Guaiane				
173. 4α,7α,Epoxyguaiane-10α,11-diol	C_15_H_26_O_3_	*S. glabra*, whole plant	EtOAc	[36]
Cadinane				
174. α-Cadinene	C_15_H_24_	*S. glabra*, leaves (Penang Hill, Penang)	Essential oil	[27]
Others				
175. Nerolidol	C_15_H_26_O	*S. glabra,* aerial part	DCM, essential oil	[27, 32]
176. Dihydrovomifoliol *O*-β-D-glucopyranoside	C_19_H_32_O_8_	*S. glabra*, whole plant	Acetone, EtOAc	[16, 64]
177. α-Humulene	C_15_H_24_	*S. glabra*, leaves (Penang Hill, Penang)	Essential oil	[27]
178. β-Caryophyllene	C_15_H_24_			
179. (*Z*,*E*)-α-Farnesene	C_15_H_24_			
180. (*E*,*E*)-α-Farnesene	C_15_H_24_			
181. (*E*,*E*)-Farnesol	C_15_H_26_O			
182. Dihydrovomifoliol	C_13_H_22_O_3_	*S. glabra*, whole plant (Jiujiang, Jiangxi province)	EtOAc	[21, 64]
183. Drovomifoliol *O*-β-D-glucopyranoside	C_19_H_30_O_8_			
184. Sarcaboside A	C_21_H_28_O_9_	*S. glabra*, whole plant (Sichuan province)	BuOH	[63]
185. Sarcaboside B	C_22_H_30_O_9_			
186. Asicariside B1 / Icariside B1	C_17_H_26_O_8_	*S. glabra*, whole plant (Jiujiang, Jiangxi province)	EtOAc	[64]
187. (*S*)-Abscisic acid	C_15_H_20_O_4_			
188. β-D-Glucopyranosyl abscisate	C_21_H_30_O_9_

Apart from the aforementioned sesquiterpenoid subtypes, there are more than a dozen other sesquiterpenoids isolated from *S. glabra* (Table 8, Fig. 8). These include aliphatic farnesenes (**175**, **179**–**181**), cyclic farnesenes (**177** and **178**), megastigmane-type sesquiterpenoids (**182** and **187**), and their glucosides (**176**, **183**, **186**, **188**). Li et al. [63] reported the isolation of two new compounds, sarcabosides A and B (**184** and **185**), from *S. glabra* specimens collected from Sichuan province. Both compounds possess interesting, highly conjugated skeletons.

#### Monoterpenoids

**Fig. 9:**
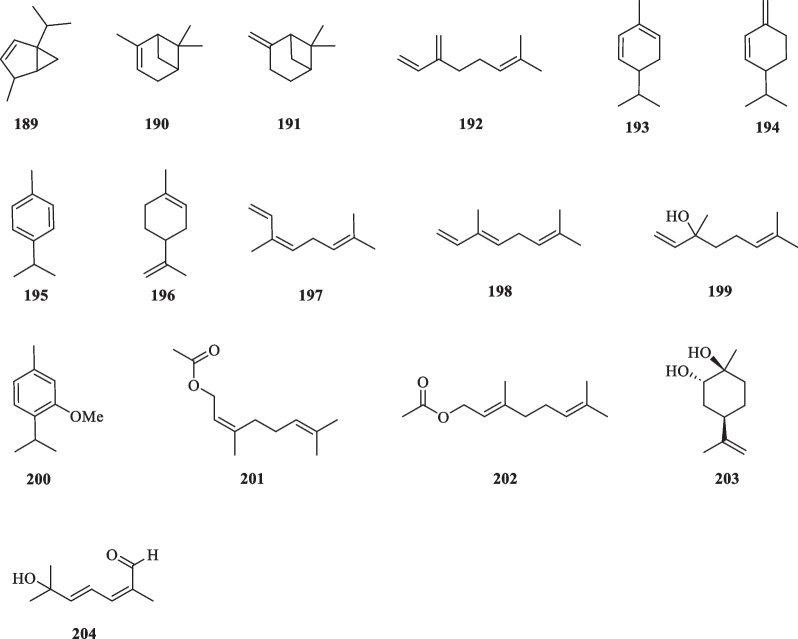
Monoterpenoids (**189**–**204**)

Monoterpenoids are a terpenoid class consisting of two isoprene units with the general molecular formula C_10_H_16_ [65]_._ They are derived from the putative precursor, geranyl diphosphate, and may exist in linear forms (acyclic) or comprise ring structures (cyclic). Acyclic monoterpenoids are formed by the head-to-tail polymerisation of isoprene monomers, whereas bicyclic monoterpenoids are formed from additional cyclisation and rearrangement via monoterpene synthases [66]. To date, 16 monoterpenoids (**189**–**204**) have been reported from the essential oils, whole plants, and seeds of *S. glabra* (Table 9, Fig. 9). Among them, seven are acyclic (**192**, **197**–**199**, **201**–**202**, **204**), six are monocyclic (**193**–**196**, **200**, **203**), and three have bicyclic structures (**189**–**191**). 6-Hydroxy-2,6-dimethylhepta-2,4-dienal (**204**) is a nine-carbon normonoterpene newly isolated from *S. glabra* [38].

**Table 9:** Monoterpenoids from *S. glabra*

Monoterpenoids	Molecular formula	Source	Fraction	References
189. α-Thujene	C_10_H_16_	*S. glabra,* leaves (Penang Hill, Penang)	Essential oil	[27]
190. α-Pinene	C_10_H_16_			
191. β-Pinene	C_10_H_16_			
192. Myrcene	C_10_H_16_			
193. α-Phellandrene	C_10_H_16_			
194. β-Phellandrene	C_10_H_16_			
195. p-Cymene	C_10_H_14_			
196. Limonene	C_10_H_16_			
197. (*Z*)-β-Ocimene	C_10_H_16_			
198. (*E*)-β-Ocimene	C_10_H_16_			
199. Linalool	C_10_H_18_O			
200. Methylthymol / 3-methoxy-p-cymene	C_11_H_16_O			
201. Neryl acetate	C_12_H_20_O_2_			
202. Geranyl acetate	C_12_H_20_O_2_			
203. (1*S*,2*S*,4*R*)-Limonene-1,2-diol	C_10_H_18_O_2_	*S. glabra*, whole plant	EtOAc	[36]
204. 6-Hydroxy-2,6-dimethylhepta-2,4-dienal	C_9_H_14_O_2_	*S. glabra,* seeds	PE	[38]

#### Diterpenoids and triterpenoids

**Fig. 10:**
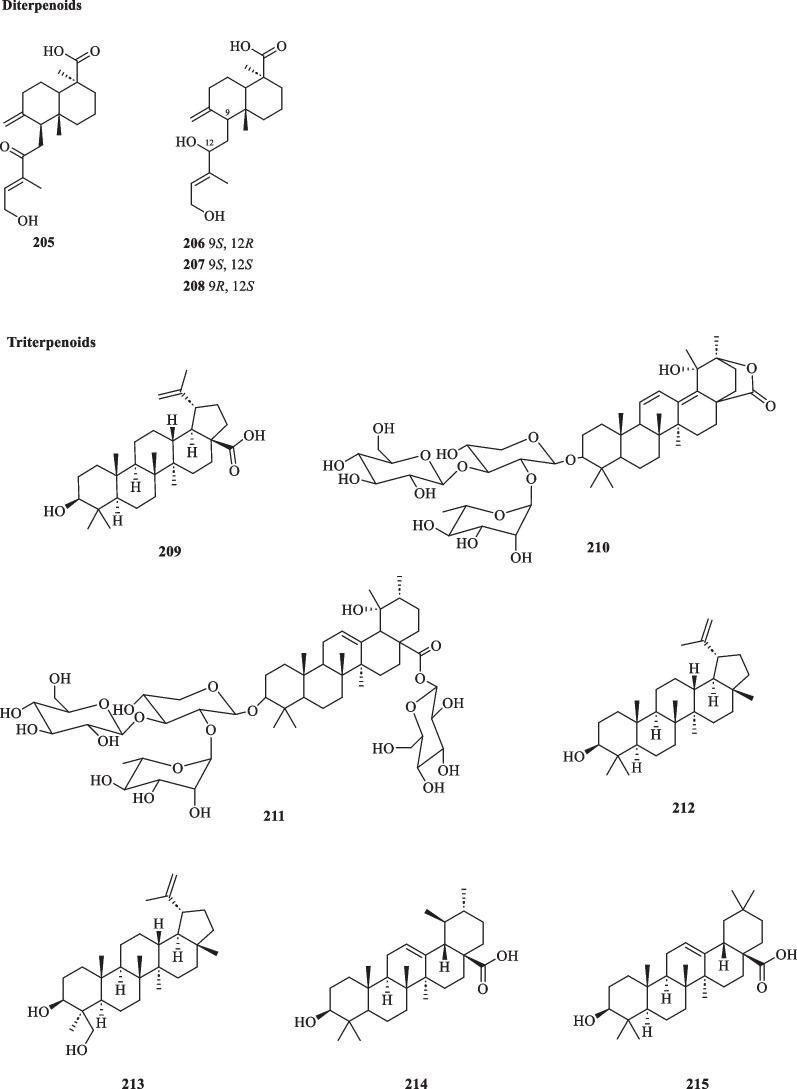
Diterpenoids (**205**–**208**) and triterpenoids (**209**–**215**)

The molecular formula of diterpenoids (C_20_H_32_) is indicative of their 20-carbon skeleton derived from the condensation of four isoprene units [65]. All diterpenoids originate from a common substrate, geranylgeranyl diphosphate, of which cyclisation into different scaffolds by diterpene synthase gives rise to their structural diversity [67]. All four reported diterpenoids (**205**–**208**) from *S. glabra* are labdane-type diterpenoids, three of which (**206**–**208**) were isolated as diastereomers (Table 10, Fig. 10).

**Table 10:** Diterpenoids and triterpenoids from *S. glabra*

Chemical constituents	Molecular formula	Source	Fraction	References
Diterpenoids				
205. 15-Hydroxy-12-oxolabda-8-(17),13*E*-dien-19-oicacid	C_20_H_30_O_4_	*S. glabra*, whole plant	EtOH	[46]
206. 12*R*,15-Dihydroxylabda-8 (17),13*E*-dien-19-oicacid	C_20_H_31_O_4_			
207. 12*S*,15-Dihydroxylabda-8 (17),13*E*-dien-19-oicacid	C_20_H_31_O_4_			
208. 9*R*,12*S*,15-Dihydroxylabda-8 (17),13*E*-dien-19-oic acid	C_20_H_31_O_4_			
Triterpenoids				
209. Betulinic acid	C_30_H_48_O_3_	*S. glabra,* aerial part	DCM	[32]
210. Sarcandroside A	C_47_H_72_O_17_	*S. glabra,* whole plant (Chongyi county, Jiangxi province)	BuOH	[69]
211. Sarcandroside B	C_53_H_86_O_22_			
212. Lupeol	C_30_H_50_O	*S. glabra,* whole plant	EtOH	[53]
213. 24-Hydroxylupeol	C_30_H_50_O_2_			
214. Ursolic acid	C_30_H_48_O_3_	*S. glabra,* whole plant	EtOH	[70]
215. Oleanolic acid	C_30_H_48_O_3_

Composed of six isoprene units (C_30_H_48_), triterpenoids have a common acyclic biosynthetic precursor, squalene [68]. Overall, their skeletons may be categorised based on the number of rings present in the structure. Pentacyclic triterpenoids are mostly prevalent in the whole plants of *S. glabra* (Table 10, Fig. 10). Seven of these triterpenoid compounds (**209**–**215**), including two new triterpenoid saponins, sarcandrosides A and B (**210** and **211**), were isolated from the aerial parts and whole plants of *S. glabra.*

#### Meroterpenoids

**Fig. 11:**
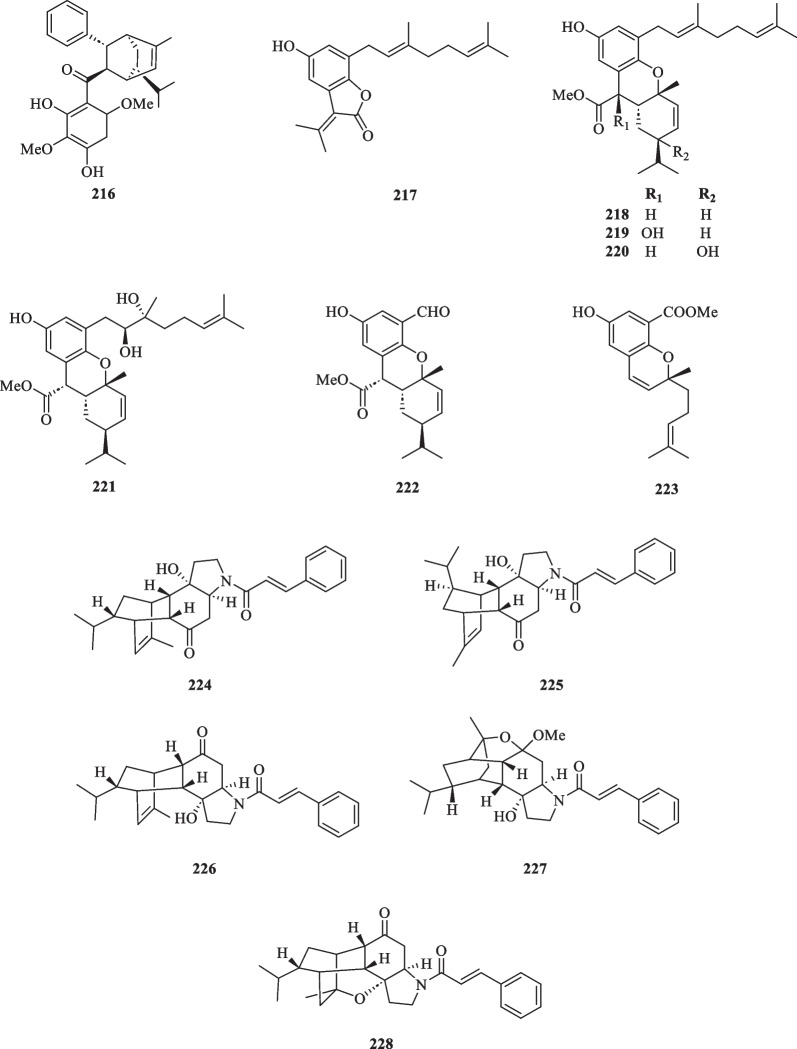
Meroterpenoids (**216**–**228**)

The term meroterpenoid was initially coined by Cornforth [71] and is used to define natural products of mixed biosynthetic origin that are partially derived from terpenoid pathways. Presently, a total of 13 meroterpenoids (**216**–**228**) have been isolated from *S. glabra* (Table 11, Fig. 11). Yang et al. [72] reported the discovery of three novel meroterpenoids, namely a chalcone-coupled monoterpenoid, glabralide A (**216**), and two geranylated meroterpenoids, glabralides B and C (**217** and **218**), from the whole plants of *S. glabra*. Compound **216** contains a bicyclo[2.2.2]octene core, while compound **218** displays an unprecedented linearly fused 6/6/6 ring system. Subsequently, a study on a *S. glabra* species from Anhui province [49] led to the isolation of five new glabralide meroterpenoids, glabralides D-H (**219**–**223**), possessing structures resembling that of **218**. Sarglamides A-E (**224**–**228**) represent five unprecedented indolidinoid-monoterpenoid hybrids derived from toussaintine C and α-phellandrene isolated from the whole plant of *S. glabra* subp. *brachystachys* [73].

**Table 11:** Meroterpenoids from *S. glabra*

Meroterpenoids	Molecular formula	Source	Fraction	References
216. Glabralide A	C_27_H_32_O_5_	*S. glabra,* whole plant (Kunming, Yunnan province)	EtOH	[72]
217. Glabralide B	C_21_H_26_O_3_			
218. Glabralide C	C_29_H_40_O_4_			
219. Glabralide D	C_29_H_40_O_5_	*S. glabra*, whole plant (Bozhou, Anhui province)	EtOAc	[49]
220. Glabralide E	C_29_H_40_O_5_			
221. Glabralide F	C_29_H_42_O_6_			
222. Glabralide G	C_20_H_24_O_5_			
223. Glabralide H	C_19_H_24_O_4_			
224. Sarglamide A	C_27_H_33_NO_3_	*S. glabra* subsp. *brachystachys*, whole plant (Wuzhi, Hainan province)	EtOAc	[73]
225. Sarglamide B	C_27_H_33_NO_3_			
226. Sarglamide C	C_27_H_33_NO_3_			
227. Sarglamide D	C_28_H_37_NO_4_			
228. Sarglamide E	C_27_H_33_NO_3_

### Isolation of phenylpropanoids from *S. glabra*

Phenylpropanoids are a vast and structurally diverse group of natural products in the plant kingdom. They play vital roles in plant development through their interaction with the environment and other living organisms. Phenylpropanoids are derived from the aromatic amino acids, phenylalanine and tyrosine, via the shikimate pathway [74]. 4-Coumaroyl-CoA represents a crucial enzyme in the metabolic network that leads to the biosynthesis of phenylpropanoid compounds such as coumarins, lignins, flavonoids, and phenolic acids [75]. Diversification of the subsets of phenylpropanoids is achieved by an array of successive enzymatic transformations such as acylation, condensation, cyclisation, glycosylation, hydroxylation, methylation, and prenylation [76].

#### Coumarins

**Fig. 12:**
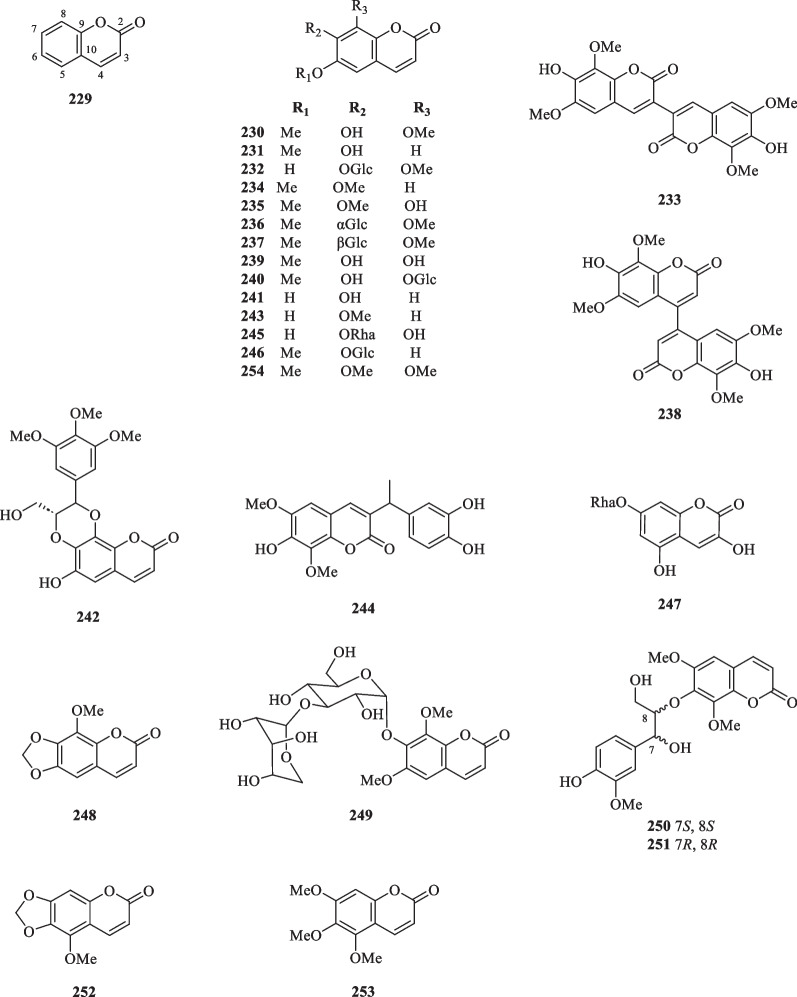
Coumarins (**229**–**254**)

Coumarins represent a class of aromatic lactones derived from the *ortho*-hydroxylated *cis*-hydroxycinnamic acid in the phenylpropanoid pathway [77]. Due to the versatility of the coumarin scaffold, various pharmacophores and functionalised coumarins could be generated via a series of transformations and substitutions, which explains the exceptional pharmacological activities displayed by this class of compounds [78–80]. Currently, the identification of 26 coumarins (**229**–**254**) has been reported in various literature of *S. glabra* (Table 12, Fig. 12), which mostly consist of known compounds, particularly isofraxidin (**230**) and its dimers (**233** an**d 238**), as well as its substituted analogues at C-6, C-7 and C-8 (**231**–**232**, **234**–**237**, **239**–**241**, **243**, **245**–**246**, **254**). This designates isofraxidin (**230**) as a representative compound of this class and establishes its inclusion as a quality control marker for medicinal preparations of *S. glabra* in the Chinese Pharmacopoeia [81]. Feng et al. [78] discovered a new coumarin, sarcandracoumarin (**244**), from the whole plant of *S. glabra* bearing a 1-phenylethyl substituent at C-3. Unfortunately, the absolute configuration of this compound remains unclear. Wang et al. [82] reported a new coumarin, 3,5-dihydroxycoumarin-7-*O*-α-L-rhamnopyranosyl-2H-chromen-2-one (**247**), possessing anti-inflammatory properties, from *S. glabra*. From the stems of *S. glabra*, Du et al. [83] uncovered the presence of five coumarins, namely, a pair of coumarin-phenylpropanoid enantiomers (7*S*,8*S*) and (7*R*,8*R*)-sarcacoumarin (**250** and **251**) with promising acetylcholinesterase inhibitory activities, and three known compounds (**252**–**254**).

**Table 12:** Coumarins from *S. glabra*

Coumarins	Molecular formula	Source	Fraction	References
229. Coumarin	C_9_H_6_O_2_	*S. glabra,* aerial part	DCM	[32]
230. Isofraxidin	C_11_H_10_O_5_	*S. glabra,* whole plant	DCM, H_2_O, EtOH	[32, 84, 85]
231. Scopoletin	C_10_H_8_O_4_	*S. glabra,* whole plant	DCM	[32, 86]
232. Eleutheroside B_1_	C_17_H_20_O_10_	*S. glabra,* whole plant	EtOH	[53]
233. 3,3′-Biisofraxidin	C_22_H_18_O_10_	*S. glabra,* whole plant	EtOH	[85]
234. Scoparone	C_11_H_10_O_4_	*S. glabra,* whole plant	EtOH	[85, 86]
235. Fraxidin	C_11_H_10_O_5_	*S. glabra,* whole plant	BuOH	[87]
236. Isofraxidin 7-*O*-α-D-glucopyranoside	C_17_H_20_O_10_			
237. Isofraxidin 7-*O*-β-D-glucopyranoside	C_17_H_20_O_10_			
238. 4,4′-Biisofraxidin	C_22_H_18_O_10_	*S. glabra,* whole plant	EtOH	[86]
239. Fraxetin	C_10_H_8_O_5_			
240. Fraxin	C_16_H_18_O_10_			
241. Esculetin	C_9_H_6_O_4_	*S. glabra,* whole plant	EtOH	[82, 86]
242. Hemidesmin-1	C_21_H_20_O_9_	*S. glabra,* whole plant	EtOH	[88]
243. Isoscopoletin	C_10_H_8_O_4_	*S. glabra,* whole plant	EtOH	[34]
244. Sarcandracoumarin	C_19_H_18_O_7_	*S. glabra,* whole plant (Xinfeng, Jiangxi province)	EtOAc	[78]
245. 6,7,8-Trihydroxycoumarin 7-*O*-rhamnopyranoside	C_15_H_15_O_9_	*S. glabra,* whole plant (Sichuan province)	ACN	[89]
246. Scopolin	C_16_H_18_O_9_	*S. glabra,* whole plant (Jiujiang, Jiangxi province)	EtOAc	[64]
247. 3,5-Dihydroxycoumarin-7-*O*-α-L-rhamnopyranosyl-2H-chromen-2-one	C_15_H_16_O_9_	*S. glabra,* whole plant (Sichuan province)	BuOH	[82]
248. 8-Methoxy-6,7-methylenedioxycoumarin	C_11_H_8_O_5_	*S. glabra,* whole plant (Chongyi county, Jiangxi province)	CHCl_3_	[90]
249. Isofraxidin 7-*O*-β-D-xylopyranosyl(1–3)-α-D-glucopyranoside	C_22_H_28_O_14_		BuOH	
250. (7*S*,8*S*)-Sarcacoumarin	C_21_H_22_O_9_	*S. glabra,* stems (Sanming, Fujian province)	AcOEt	[83]
251. (7*R*,8*R*)-Sarcacoumarin	C_21_H_22_O_9_			
252. 5-Methoxy-6,7-methylenedioxycoumarin	C_11_H_8_O_5_			
253. 5,6,7-Trimethoxycoumarin	C_12_H_12_O_5_			
254. 6,7,8-Trimethoxycoumarin	C_12_H_12_O_5_

#### Lignans and neolignans

**Fig. 13:**
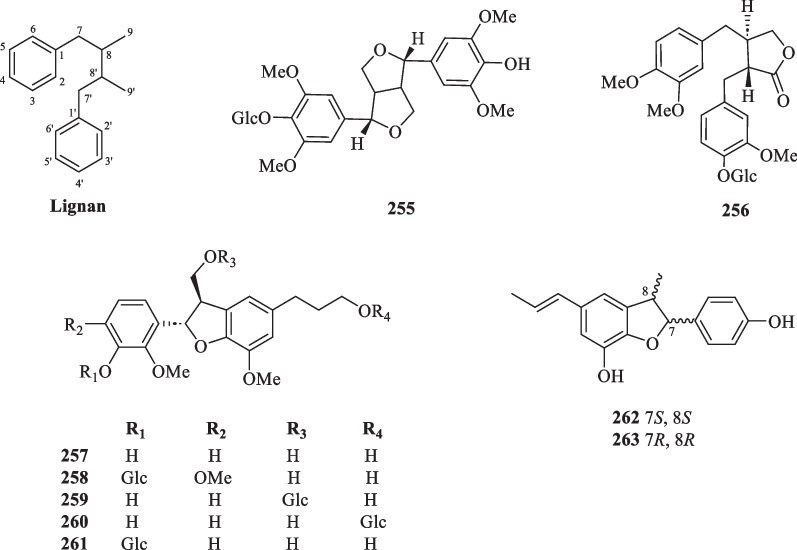
Lignans (**255** and **256**) and neolignans (**257–263**)

Lignans and neolignans are dimeric structures commonly derived from the oxidative coupling of two lignol units. They may differ in the degree of oxidation of the three-carbon sidechain and the substitution on the aromatic ring [91]. Up to the present, only two lignans (**255** and **256**) and an array of neolignans (**257**–**263**) were isolated from the whole plants of *S. glabra* (Table 13, Fig. 13). The isolation of a pair of dihydrobenzofuran neolignan enantiomers, (+) and (−)-sarcanan A (**262** and **263**), from the aerial parts of *S. glabra* was recently reported [79].

**Table 13:** Lignans and neolignans from *S. glabra*

Lignans and neolignans	Molecular formula	Source	Fraction	References
255. Syringaresinol monoside	C_28_H_36_O_13_	*S. glabra,* whole plant	EtOH	[34]
256. Styraxiaponoside B	C_27_H_34_O_6_			
257. (−)-(7*S*,8*R*)-Dihydrodehydrodiconiferyl alcohol	C_20_H_24_O_6_	*S. glabra,* whole plant	EtOH, EtOAc	[79, 88]
258. (−)-(7*S*,8*R*)-5-Methoxy dihydrodehydrodiconiferyl alcohol 4-*O*-β-D-glucopyranoside	C_27_H_36_O_12_	*S. glabra,* whole plant	EtOAc	[79]
259. (−)-(7*S*,8*R*)-Dihydrodehydrodiconiferyl alcohol 9-*O*-α-D-glucopyranoside	C_26_H_34_O_11_			
260. (−)-(7*S*,8*R*)-Dihydrodehydrodiconiferyl alcohol 9′-*O*-α-D-glucopyranoside	C_26_H_34_O_11_			
261. (−)-(7*S*,8*R*)-Dihydrodehydrodiconiferyl alcohol 4-*O*-α-D-glucopyranoside	C_26_H_34_O_11_			
262. (+)-Sarcanan A	C_18_H_18_O_3_	*S. glabra,* aerial parts	–	[92]
263. (−)-Sarcanan A	C_18_H_18_O_3_

#### Flavonoids

Flavonoids, a diversified group of phytochemicals found in many natural sources, are the second most predominant secondary metabolites in *S. glabra*. Despite having a common structure, flavonoids in *S. glabra* have intrigued the interest of numerous scientists due to their extensive pharmacological and biological activities that hold significant pharmaceutical importance [93, 94].

Chemically, the general structure of flavonoids consists of a C6-C3-C6 diphenylpropane backbone [95]. Flavonoids can be divided into a myriad of subgroups in terms of ring arrangements and functionalisation of the ring systems. Flavonoids in which ring B is attached to the C-3 position of ring C are called isoflavonoids [96], while those in which ring B is linked to C-2 of ring C are further categorised into different subtypes depending on the hydroxylation pattern and variations in the chromane ring or ring C [97]. Some relevant examples of these subtypes include flavans, flavones, flavonols, flavanones, flavanols, flavanonols, and anthocyanins. Chalcone otherwise known as open-chain flavonoids are distinguished from other flavonoids by their two aromatic rings (A and B), which are linked by an α,β-unsaturated carbonyl chain.

##### Chalcones

**Fig. 14:**
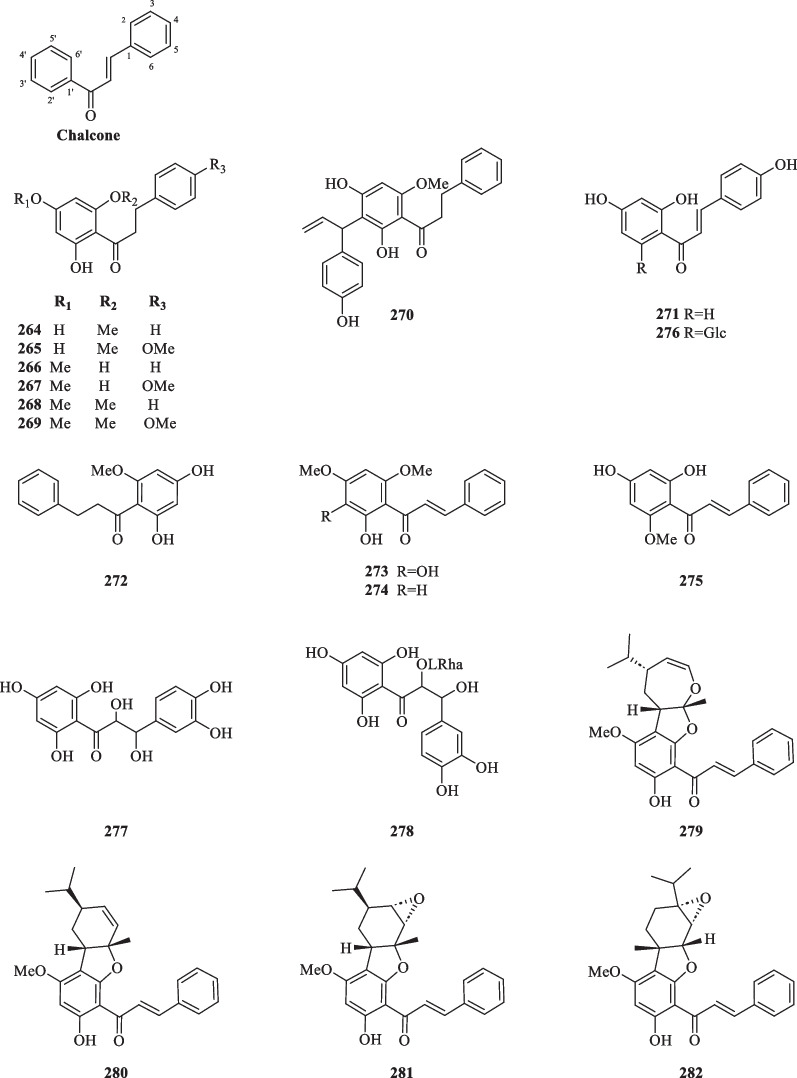

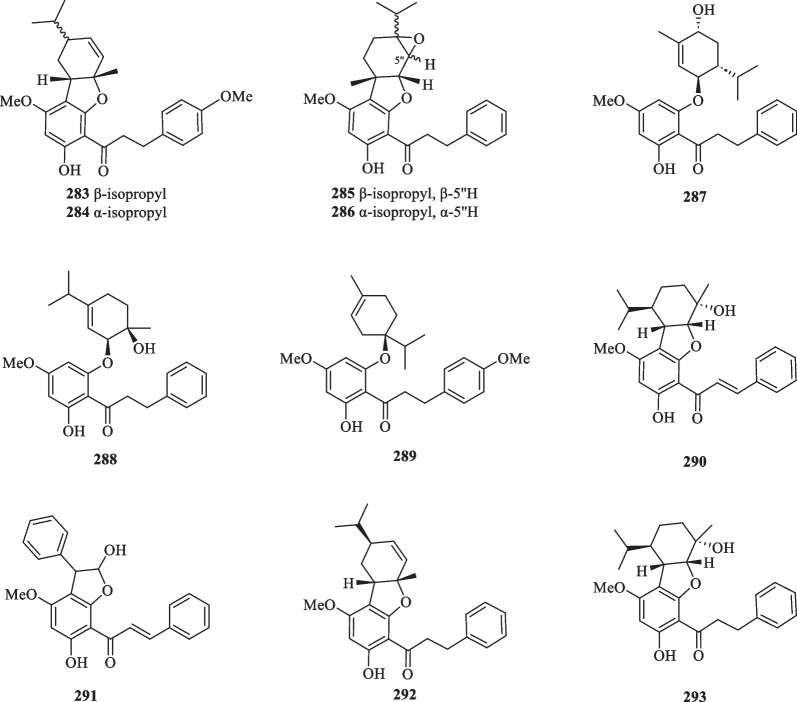
Chalcone-type flavonoids (**264**–**293**)

Following the isolation of a series of dihydrochalcones (**264**–**269**) from the aerial parts of *S. glabra,* subsequent studies have reported 21 more chalcones (**270**–**272**, **276**–**293**) from *S. glabra* and an additional three (**272**–**275**) from its subspecies *S. hainanensis* (Table 14, Fig. 14). Interestingly, Liu et al. [98] isolated a rare class of uncommon monoterpene-chalcone conjugates with potential autophagy-inducing activities from the aerial parts of *S. glabra,* some of which are connected via a dihydrofuran ring or an ether bridge. The compounds include four monoterpene-conjugated chalcones, glabratins A–D (**279**–**282**), seven monoterpene-conjugated dihydrochalcones, glabratins E-K (**283**–**289**), and four known analogues (**290**–**293**).

**Table 14:** Chalcone and anthocyanidin-type flavonoids from *S. glabra*

Chemical constituents	Molecular formula	Source	Fraction	References
Chalcones				
264. 2′,4′-Dihydroxy-6′-methoxydihydrochalcone	C_16_H_16_O_4_	*S. glabra,* aerial part	DCM	[32]
265. 2′,4′-Dihydroxy-4,6′-dimethoxydihydrochalcone	C_17_H_18_O_5_			
266. 2′,6′-Dihydroxy-4′-methoxydihydrochalcone	C_16_H_16_O_4_			
267. 2′,6′-Dihydroxy-4,4′-dimethoxydihydrochalcone	C_17_H_18_O_5_			
268. 2′-Hydroxy-4′,6′-dimethoxydihydrochalcone	C_17_H_18_O_4_			
269. 2′-Hydroxy-4,4′,6′-trimethoxydihydrochalcone	C_18_H_20_O_5_			
270. 3′-(7′′-Allylphenyl)-2′,4′,4′′-trihydroxy-6′-methoxydihydrochalcone	C_25_H_24_O_5_	*S. glabra,* whole plant	EtOH	[101]
271. Isoliquiritigenin	C_15_H_12_O_4_	*S. glabra,* whole plant	EtOH	[102]
272. Uvangoletin	C_16_H_16_O_4_	*S. glabra,* whole plant	EtOH	[85]
273. 2′,3′-Dihydroxy-4′,6′-dimethoxychalcone	C_17_H_16_O_5_	*S. hainanensis,* whole plant	PE	[103]
274. 2′-Hydroxy-4′,6′-dimethoxychalcone	C_17_H_16_O_4_			
275. Cardamonin	C_16_H_14_O_4_			
276. Isoliquiritigenin 2’-*O*-β-D-glucoside	C_21_H_23_O_10_	*S. glabra,* whole plant (Sichuan province)	ACN	[89]
277. Cilicicone B	C_15_H_14_O_8_	*S. glabra,* whole plant	MeOH	[104]
278. β,2,3′,4,4′,6-Hexahydroxy-α-(α-L-rhamnopyranosyl) dihydrochalcone	C_21_H_25_O_12_			
279. Glabratin A	C_26_H_28_O_5_	*S. glabra,* aerial part (Rong’an county, Guangxi province)	PE	[98]
280. Glabratin B	C_26_H_28_O_4_			
281. Glabratin C	C_26_H_28_O_5_			
282. Glabratin D	C_26_H_28_O_5_			
283. Glabratin E	C_27_H_32_O_5_			
284. Glabratin F	C_27_H_32_O_5_			
285. Glabratin G	C_26_H_30_O_5_			
286. Glabratin H	C_26_H_30_O_5_			
287. Glabratin I	C_26_H_32_O_5_			
288. Glabratin J	C_26_H_32_O_5_			
289. Glabratin K	C_27_H_34_O_5_			
290. Linderol A	C_26_H_30_O_5_			
291. Cathayenone A	C_24_H_20_O_5_			
292. Adunctin B	C_26_H_30_O_4_			
293. Adunctin E	C_26_H_32_O_5_			
Anthocyanidins				
294. Pelargonidin 3-rhamnosylglucoside	C_27_H_31_O_14_	*C. glaber,* fruits	–	[100]
295. Cyanidin 3-rhamnosylglucoside	C_27_H_31_O_15_			
296. (−)-Epiafzelechin 7-*O*-β-D-glucopyranoside	C_21_H_24_O_10_	*S. glabra,* whole plant	ACN	[89]

##### Anthocyanidins

**Fig. 15:**
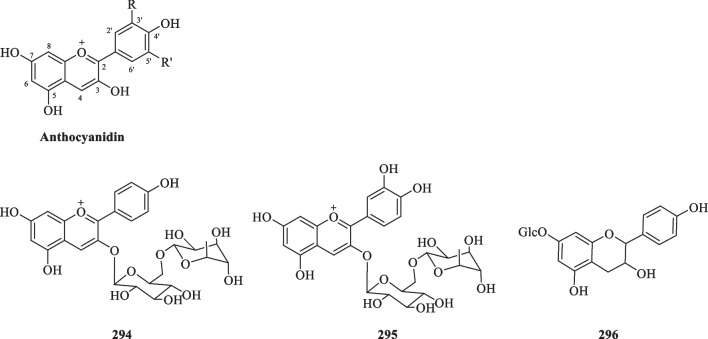
Anthocyanidin-type flavonoids (**294**–**296**)

Anthocyanidins are a class of flavonoids known for their roles as natural pigments that bestow fruits and flowers different colours. Their distinctive skeletal backbone is based on a flavylium cation that lacks the ketone group at C-4 [99] So far, only three anthocyanidin-type compounds (**294**–**296**) were characterised from *S. glabra* (Table 14, Fig. 15)*.* Ishikura [100] was the first researcher to report the occurrence of two glycosides of anthocyanidins (**294** and **295**) from the fruits of *C. glaber* (syn*. S. glabra),* whereas Li et al. [89] isolated a leucoanthocyanidin compound (**296**) from the whole plant of *S. glabra*.

##### Flavones and flavonols

**Fig. 16:**
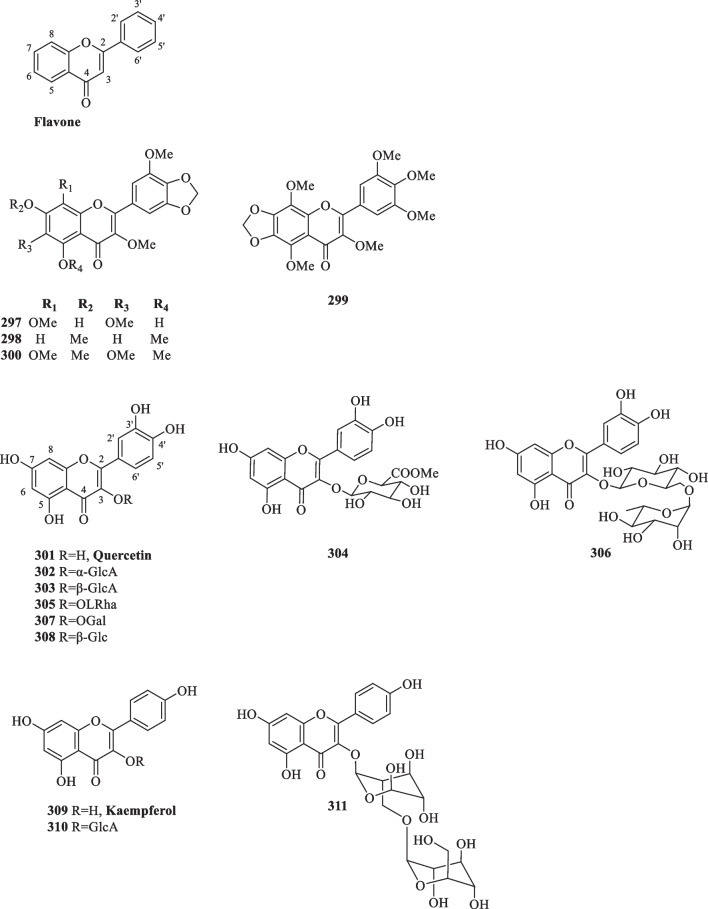
Flavones and flavonols (**297**–**311**)

The backbones of flavones and their hydroxylated derivatives (flavonols) are distinguished by the presence of a double bond between C-2 and C-3, which extends the π-conjugation onto the carbonyl group in the pyranone ring [105]. Collective studies on *S. glabra* have reported a total of 15 flavone and flavonol-type compounds (**297**–**311**), including a number of quercetin and kaempferol derivatives (Table 15, Fig. 16).

**Table 15:** Flavones and flavonols from *S. glabra*

Flavones and flavonols	Molecular formula	Source	Fraction	References
297. 5,7-Dihydroxy-3,3′,6,8-tetramethoxy-4′,5′-methylenedioxyflavone	C_20_H_18_O_10_	*S. glabra*	–	[106]
298. 3,3′,5,7-Tetramethoxy-4′,5′-methylenedioxyflavone	C_20_H_18_O_8_			
299. 3,3′,4′,5,5′,8-Hexamethoxy-6,7-methylenedioxyflavone	C_22_H_22_O_10_			
300. 3,3′,5,6,7,8-Hexamethoxy-4′,5′-methylenedioxyflavone	C_22_H_22_O_10_			
301. Quercetin	C_15_H_10_O_7_	*S. glabra,* whole plant	EtOH	[102]
302. Quercetin 3-*O*-α-D-glucuronide	C_21_H_18_O_13_	*S. glabra,* whole plant	H_2_O	[109]
303. Quercetin 3-*O*-β-D-glucuronide	C_21_H_18_O_13_			
304. Quercetin 3-*O*-β-D-glucuronopyranoside methyl ester	C_22_H_20_O_13_			
305. Quercetin 3-*O*-*a*-L-rhamnoside	C_21_H_20_O_11_	*S. glabra,* whole plant	EtOH	[110]
306. Quercetin 3-*O*-β-D-rutinoside	C_27_H_30_O_16_	*S. glabra,* whole plant	EtOH	[70, 110]
307. Hyperoside	C_21_H_20_O_12_	*S. glabra,* whole plant	EtOH	[70]
308. Quercetin 3-β-glucoside	C_21_H_20_O_12_	*S. glabra*	–	[106]
309. Kaempferol	C_15_H_10_O_6_	*S. glabra, S. hainanensis,* whole plant	BuOH, EtOAc	[87, 111]
310. Kaempferol 3-*O*-β-D-glucuronide	C_21_H_18_O_12_	*S. glabra, S. hainanensis,* whole plant	H_2_O, EtOAc	[109, 111]
311. Kaempferol 3-*O*-rhamnopyranosyl (1→6) glucopyranoside	C_27_H_30_O_15_	*S. glabra,* whole plant (Chongyi county, Jiangxi province)	BuOH	[90]

In a recent study, Qin et al. [106] revealed the presence of two new methylenedioxyflavones (**297** and **298**) and two known derivatives (**299** and **300**) from *S. glabra,* among which compounds **297**, **299** and **300** showed pronounced cytotoxic effects. Ubiquitous in various natural sources, the structure of quercetin (**301**) comprises a pentahydroxyflavone, which is oftentimes conjugated with residual sugars to form quercetin glycosides [107]. Overall, all reported quercetin-type flavonoids from *S. glabra* were obtained from the whole plants and are derivatives substituted with glycoside groups at C-3, such as glucuronide (**302**–**304**), rhamnoside (**305**), rutinoside (**306**), galactose (**307**) and glucoside (**308**). Kaempferol (**309**) is a tetrahydroxyflavone in which the four hydroxyl groups are at C-3, C-5, C-7 and C-4′ [108]. So far, only three compounds of this class (**309**–**311**) have been isolated from the whole plants of *S. glabra* and *S. hainanensis* (subsp. *brachystachys*).

##### Flavanones and flavanols

**Fig. 17:**
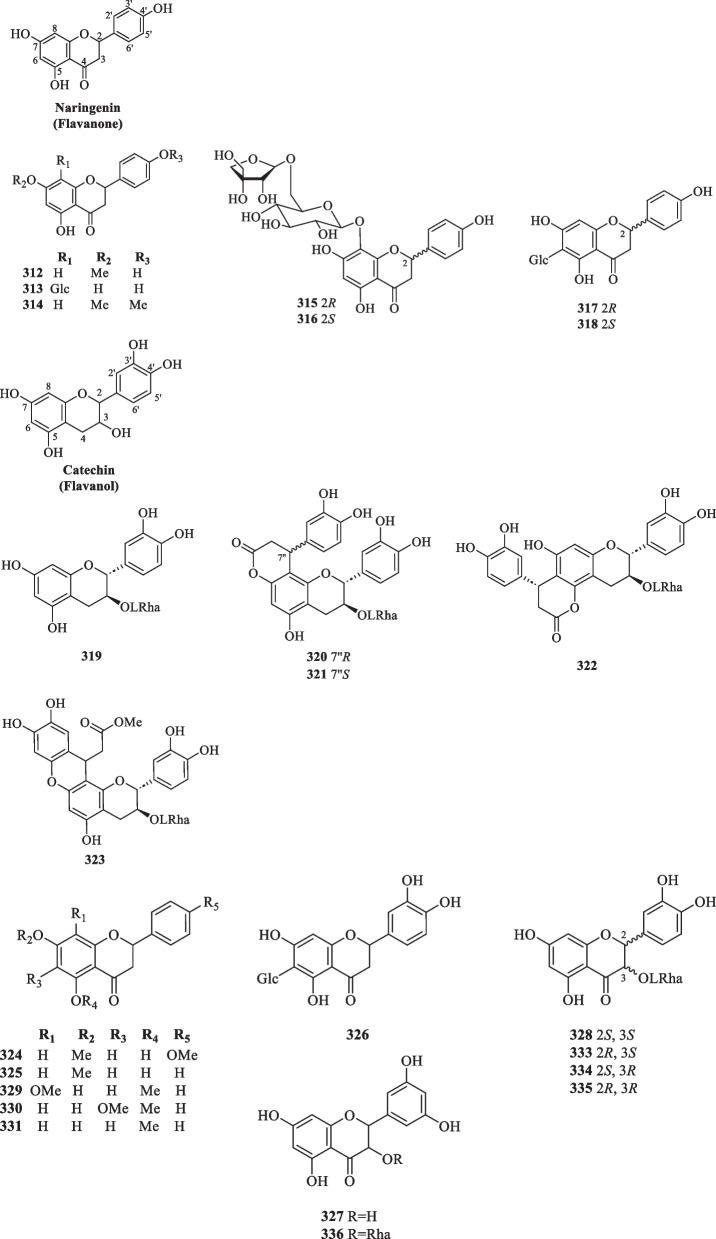

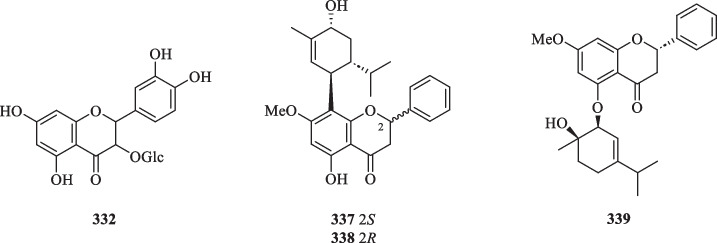
Flavanones and flavanols (**312**–**339**)

Derived from flavones, flavanones are important precursors and key intermediates in the flavonoid biosynthetic pathway. Flavanones and their hydroxylated derivatives (flavanols) differ from flavones and flavonols by two features: the presence of a C-2 chiral centre and the absence of a double bond between C-2 and C-3 [112]. Up to now, a total of 29 flavanone and flavanol-type compounds (**312**–**339**), including naringenin and catechin derivatives, were reported from *S. glabra* (Table 16, Fig. 17).

**Table 16:** Flavanones, flavanols, and dimeric flavonoids from *S. glabra*

Chemical constituents	Molecular formula	Source	Fraction	References
Flavanones and flavanols				
312. 7-Methylnaringenin	C_16_H_14_O_5_	*S. glabra,* whole plant	EtOH	[53]
313. 5,7,4′-Trihydroxy 8-C-β-D-glucopyranosyl flavanone/ naringenin 8-C-β-D-glucopyranoside	C_21_H_20_O_11_	*S. glabra,* whole plant	H_2_O	[109]
314. Naringenin-4′,7-dimethyl ether	C_17_H_16_O_5_	*S. hainanensis,* whole plant	DCM	[111]
315. (2*R*)-Naringenin 8-C-β-D-glucopyranosyl-(6 → 1)-apiose	C_26_H_29_O_14_	*S. glabra,* whole plant (Sichuan province)	ACN	[89]
316. (2*S*)-Naringenin 8-C-β-D-glucopyranosyl-(6 → 1)-apiose	C_26_H_29_O_14_			
317. (2*R*)-Naringenin 6-C-β-D-glucopyranoside	C_21_H_21_O_10_	*S. glabra,* whole plant, aerial part	H_2_O	[114]
318. (2*S*)-Naringenin 6-C-β-D-glucopyranoside	C_21_H_21_O_10_			
319. Catechin 3-*O*-α-L-rhamnopyranoside	C_21_H_24_O_10_	*S. glabra,* whole plant	EtOH	[59]
320. Glabraoside A	C_30_H_30_O_13_	*S. glabra,* whole plant	EtOH	[101]
321. Glabraoside B	C_30_H_30_O_13_	*S. glabra,* whole plant	EtOH	[59]
322. Glabraoside C	C_30_H_30_O_13_	*S. glabra,* whole plant	EtOH	[20]
323. Glabraoside D	C_31_H_32_O_14_			
324. 5-Hydroxy-7,4′-dimethoxydihyflavanone	C_17_H_16_O_5_	*S. glabra,* aerial part and whole plant	DCM	[32, 53, 85]
325. 5-Hydroxy-7-methoxyflavanone	C_16_H_14_O_4_	*S. glabra, S.hainanensis* aerial part, whole plant	DCM, EtOH, BuOH	[32, 53, 87, 116]
326. 5,7,3′,4′-Tetrahydroxy 6-C-β-D-glucopyranosyl flavanone	C_21_H_21_O_11_	*S. glabra,* whole plant	BuOH	[87]
327. (+)-3,3′,5,5′,7-Pentahydroxyflavonone	C_15_H_11_O_7_	*S. glabra,* whole plant	EtOH	[88]
328. Neoastilbin	C_21_H_22_O_11_	*S. glabra,* whole plant	H_2_O, EtOH	[34, 109]
329. 7-Hydroxy-5,8-dimethoxyflavanone	C_17_H_16_O_5_	*S. hainanensis,* whole plant	DCM	[116]
330. 7-Hydroxy-5,6-dimethoxyflavanone	C_17_H_15_O_5_	*S. hainanensis,* whole plant	DCM, EtOAc	[111]
331. 7-Hydroxy-5-methoxyflavanone	C_16_H_14_O_4_			
332. 5,7,3′,4′-Tetrahydroxyflavanone 3-*O*-glucoside	C_21_H_22_O_12_			
333. Isoastilbin	C_21_H_22_O_11_	*S. glabra,* whole plant	EtOH	[34]
334. Neoisoastilbin	C_21_H_22_O_11_			
335. Astilbin	C_21_H_22_O_11_			
336. 3,3′,5,5′,7-Pentahydroxyflavonone 3-*O*-α-L-rhamnopyranoside	C_21_H_21_O_11_	*S. glabra,* whole plant (Sichuan province)	ACN	[89]
337. Glabratin L	C_26_H_30_O_5_	*S. glabra,* aerial part (Rong’an county, Guangxi province)	PE	[98]
338. Glabratin M	C_26_H_30_O_5_			
339. Glabratin N	C_26_H_30_O_5_			
Dimeric flavonoids				
340. Sarcandrone A	C_33_H_30_O_8_	*S. hainanensis,* whole plant	DCM	[116]
341. Sarcandrone B	C_33_H_30_O_8_			
342. Sarcandrone C	C_33_H_30_O_8_	*S. hainanensis,* whole plant	DCM	[111]
343. Sarcandrone D	C_33_H_30_O_8_

Naringenin is a pertinent example of a flavanone aglycone having three hydroxyl groups at C-5, C-7, and C-4' [113]. Most of the compounds belonging to this class occur as methoxy and glycosidic derivatives (**312**–**314**) and were isolated from the whole plants of *S. glabra* and *S. hainanensis* (subsp.). Interestingly, some of the naringenin-type compounds isolated from *S. glabra* are epimeric at C-2, as exemplified by **315**–**318** [89, 114].

Catechins are secondary metabolites that contribute to the antioxidative activities in plants [115]. They exist as minor constituents in *S. glabra* and are flavan-3-ols characterised by the absence of a ketone group at the C-4 position. To date, only six catechin-type flavanols (**319**–**323**) were documented in the literature. Li [59] reported the isolation of a rhamnopyranoside derivative (**319**) and two new epimeric phenylpropanoid-substituted catechin glycosides, glabraosides A and B (**320** and **321**), from the whole plant of *S. glabra.* Subsequently, Wang et al. [20] expanded the family by reporting two new catechin glycosides, glabraosides C and D (**322** and **323**), with their phenylpropanoid fragments fused to ring A at C-5/C-6 and C-7/C-8, respectively. Liu et al. [98] reported glabratins L-N (**337**–**339**) as three monoterpene-flavanone conjugates from the aerial parts of *S. glabra.* Compounds **337** and **338** were obtained as a pair of C-2 epimers, whereas the flavanone core of **339** is linked to the monoterpene unit via an ether bridge instead of a C–C bond.

##### Dimeric flavonoids

**Fig. 18:**
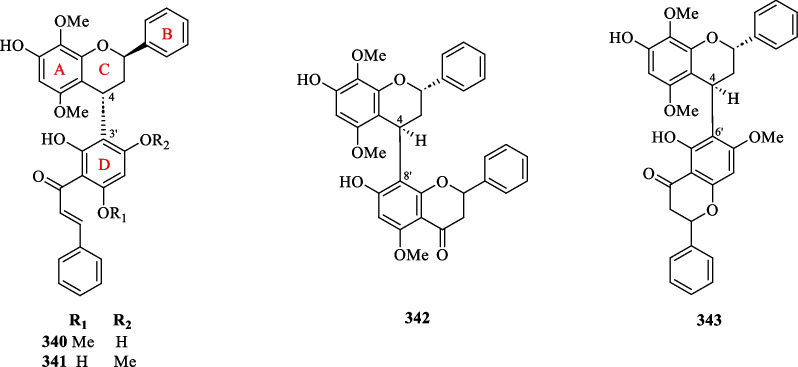
Dimeric flavonoids (**340**–**343**)

The occurrence of dimeric flavonoids was found to be exclusive to the *S. hainanensis* subspecies, as evidenced by the isolation of four compounds (**340**–**343**) from the dichloromethane extract of the whole plant (Table 16, Fig. 18). Sarcandrones A and B (**340** and **341**) represent new hybrid flavan-chalcones that are linked from C-4 of the flavan moiety to C-3′ of the chalcone moiety [116]. On the other hand, sarcandrones C and D (**342** and **343**) were characterised as analogues that are linked from the flavan moiety to the flavanone moiety at C-4 to C-8′ and C-4 to C-6′, respectively [111].

### Isolation of anthraquinones, organic acids, and organic esters from *S. glabra*

**Fig. 19:**
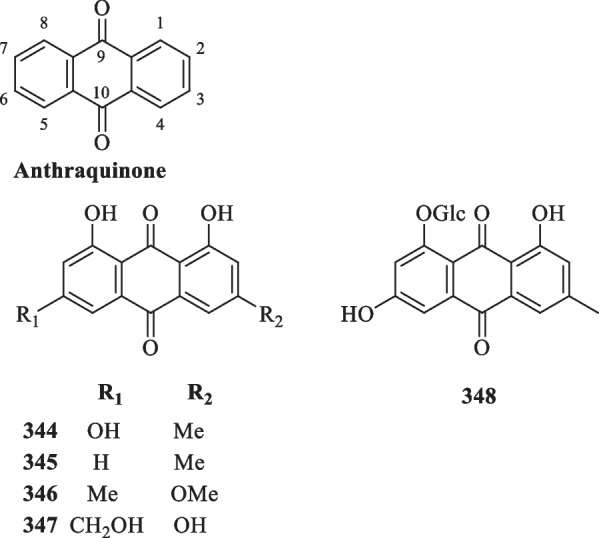
Anthraquinones (**344**–**348**)

Anthraquinones, also referred to as anthracenediones or dioxoanthracenes, are associated with the quinone class under the polyketide family. A characteristic feature of anthraquinones is their polycyclic aromatic structure wherein two keto groups are located on the central ring [117]. Current literature on *S. glabra* has cumulatively reported five anthraquinone-type compounds (**344**–**348**), which mainly consist of hydroxy, methoxy or glycoside derivatives (Table 17, Fig. 19).

**Table 17:** Anthraquinones, organic acids, and organic esters from *S. glabra*

Chemical constituents	Molecular formula	Source	Fraction	References
Anthraquinones				
344. Emodin	C_15_H_10_O_5_	*S. hainanensis, S. glabra,* whole plant	PE, EtOH	[103, 119]
345. Chrysophanol	C_15_H_10_O_4_	*S. hainanensis, S. glabra,* whole plant	PE, EtOH	[70, 103]
346. Physcion	C_16_H_12_O_5_	*S. glabra,* whole plant	EtOH	[119]
347. Citreorosein	C_15_H_10_O_6_	*S. glabra,* whole plant	EtOH	[70]
348. Emodin 8-*O*-β-D-glucopyranoside	C_21_H_20_O_10_			
Organic acids				
349. Fumaric acid	C_4_H_3_O_4_	*S. glabra,* whole plant	H_2_O	[120, 121]
350. Stearic acid	C_18_H_36_O_2_	*S. glabra, S. hainanensis,* whole plant	EtOH, PE	[103, 122]
351. Palmitic acid	C_16_H_32_O_2_	*S. glabra, S. hainanensis,* whole plant	EtOH, PE	[53, 85, 103]
352. 3,4-Dihydroxybenzoic acid / protocatechuic acid	C_7_H_5_O_4_	*S. glabra,* whole plant	EtOH, H_2_O, EtOAc	[53, 78, 84, 109]
353. Ferulic acid	C_10_H_10_O_4_	*S. glabra,* whole plant	EtOH	[59]
354. *N*-Pentadecanoic acid	C_15_H_30_O_2_	*S. glabra,* whole plant	EtOH	[85]
355. Caffeic acid	C_9_H_8_O_4_	*S. glabra,* whole plant	H_2_O	[84]
356. Icosanoic acid	C_20_H_40_O_2_	*S. hainanensis,* whole plant	PE	[103]
357. Isovanillic acid	C_8_H_8_O_4_	*S. glabra,* stems	H_2_O	[123]
358. Vanillic acid	C_8_H_8_O_4_	*S. glabra,* whole plant	EtOAc	[78, 124]
359. Syringic acid	C_9_H_10_O_5_	*S. glabra,* whole plant	EtOAc	[78]
360. *O*-Phthalic acid	C_8_H_6_O_4_	*S. glabra,* whole plant		[110]
361. *N*-Docosanoic acid	C_22_H_44_O_2_			
362. *N*-heptadecanoic acid	C_17_H_34_O_2_			
363. Tetracosanoic acid	C_24_H_48_O_2_			
364. Succinic acid	C_4_H_6_O_4_			
365. *p*-Hydroxybenzoic acid	C_7_H_6_O_3_	*S. glabra,* whole plant	H_2_O	[110, 124]
366. Quinic acid	C_7_H_12_O_6_	*S. glabra,* whole plant (Sichuan province)	ACN	[89]
367. Glucosyringic acid	C_15_H_20_O_10_	*S. glabra,* whole plant	EtOAc	[79]
Organic esters				
368. Methyl α,3,4-trihydroxybenzenepropanoate	C_10_H_12_O_5_	*S. glabra,* whole plant	EtOH	[59]
369. Rosmarinic acid	C_18_H_16_O_8_	*S. glabra,* whole plant	H_2_O	[84]
370. Methyl rosmarinate	C_19_H_18_O_8_			
371. Dibutyl phthalate	C_16_H_22_O_4_			
372. Methyl 5-*O*-caffeoylquinilic acid	C_17_H_20_O_9_	*S. glabra,* whole plant	H_2_O	[109]
373. Ethyl rosmarinate	C_20_H_20_O_8_	*S. glabra,* whole plant	EtOH	[88]
374. 2-Methylbutyl 2-methylbutyrate	C_10_H_20_O_2_	*S. glabra,* leaves (Penang Hill, Penang)	Essential oil	[27]
375. Octyl acetate	C_10_H_20_O_2_			
376. 3-Methylbutyl hexanoate	C_11_H_22_O_2_			
377. 3-*O*-Caffeoylquinic acid / chlorogenic acid	C_16_H_18_O_9_	*S. glabra,* whole plant	H_2_O	[125]
378. 3-*O*-Caffeoylquinic acid methyl ester	C_17_H_20_O_9_			
379. 4-*O*-Caffeoylquinic acid	C_16_H_18_O_9_			
380. 4-*O*-Caffeoylquinic acid methyl ester	C_17_H_20_O_9_			
381. 5-*O*-Caffeoylshikimic acid	C_16_H_16_O_8_	*S. glabra,* whole plant	EtOH, EtOAc	[34, 79]
382. Rosmarinic acid 4-*O*-β-D glucoside	C_24_H_26_O_13_	*S. glabra,* stems	H_2_O	[123]
383. 3-*O*-Caffeoylshikimic acid	C_16_H_16_O_8_	*S. glabra,* whole plant	BuOH,	[78]
384. 4-*O*-Caffeoylshikimic acid	C_16_H_16_O_8_			
385. 5-*O*-Caffeoylquinic acid	C_16_H_18_O_9_			
386. Caffeic acid ethyl ester / ethyl caffeate	C_11_H_12_O_4_	*S. glabra,* whole plant	EtOH	[126]
387. Vinyl caffeate	C_11_H_10_O_4_			
388. Neochlorogenic acid	C_16_H_18_O_9_			
389. Cryptochlorogenic acid	C_16_H_18_O_9_			
390. Benzyl 2-β-glucopyranosyloxybenzoate	C_20_H_22_O_8_	*S. glabra,* whole plant (Jiujian, Jiangxi province)	EtOAc	[79]
391. 3,4-Dihydroxyphenethyl caffeate	C_17_H_16_O_6_	*S. glabra,* whole plant	EtOH	[90]

**Fig. 20:**
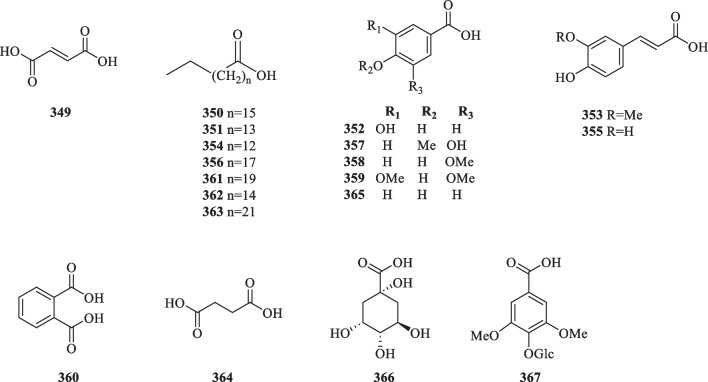
Organic acids (**349**–**367**)

As shown in Table 17 and Fig. 20*, S. glabra* plants are rich sources of organic acids. At present, more than a dozen known organic acids have been isolated from *S. glabra* (**349**, **352**–**355**, **357**–**367**), while three (**350**–**351**, **356**) were obtained from the subspecies *S. hainanensis*. These include dicarboxylic acids (**349** and **364**), long-chained fatty acids (**350**, **351**, **354**, **356**, **361**–**363**), and multi-substituted phenolic acids (**352**–**353**, **355**, **357**–**360**, **364**–**367**).

**Fig. 21:**
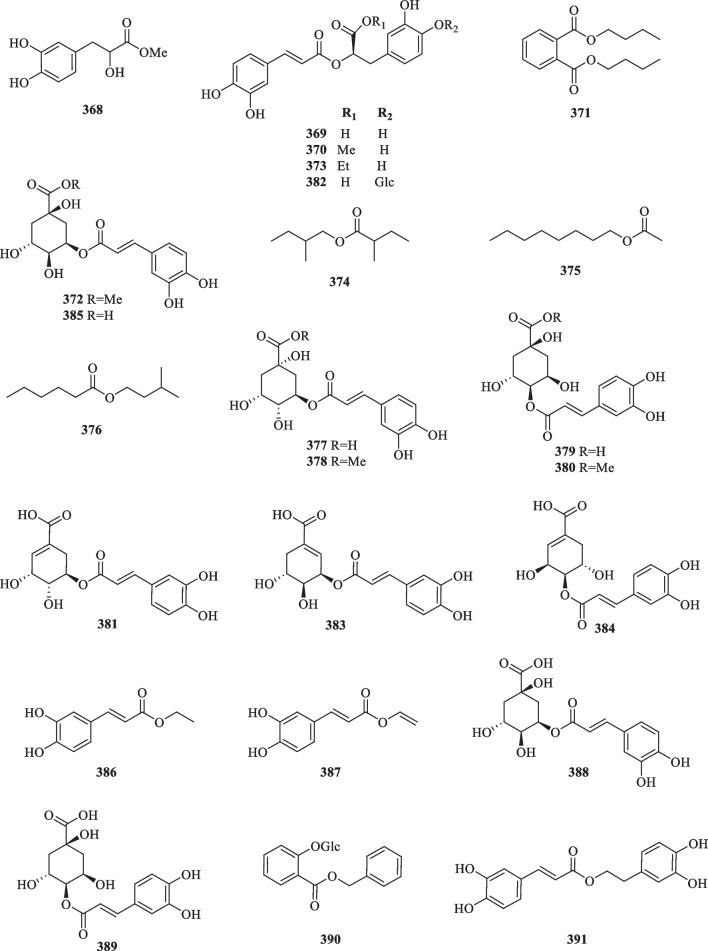
Organic esters (**368**–**391**)

Organic esters (**368**–**391**) were particularly abundant in *S. glabra*. These compounds are distributed in different plant parts and their structures could be divided into several categories (Table 17, Fig. 21). For instance, compounds **386**, **387**, and **391** were classified as caffeic acid derivatives, while compounds **381**, **383**, and **384** are generally known as caffeoylshikimic acids. Esters in which their quinic acid core is acylated with one or more caffeoyl groups are known as caffeoylquinic acids or chlorogenic acids. Eight compounds of this group (**372**, **377**–**380**, **385**, **388**–**389**) were isolated from the polar partitions of *S. glabra*. From the whole plant of *S. glabra*, Wu et al. [79] reported a new glycoside compound (**390**), benzyl 2-β-glucopyranosyloxybenzoate. Among all organic esters, rosmarinic acid (**369**) is specifically recognised for its pronounced pharmacological activities and its high content within *S. glabra* [3, 118]. For this reason, rosmarinic acid is established as one of the key quality control markers in the Chinese Pharmacopoeia [81].

Interestingly, the distribution of organic esters differs between the plant parts of *S. glabra*. This hypothesis was validated by Zhou et al. [114] who concluded that chlorogenic acids, caffeic acids, and 4-*O*-glucopyranosyl rosmarinic acid were mostly concentrated in the stems, whereas chlorogenic acids were dominant in the leaves. On the other hand, rosmarinic acids were found to be distributed in the aerial parts (stems and leaves) of *S. glabra*.

### Isolation of alcohols and sterols from *S. glabra*

**Fig. 22:**
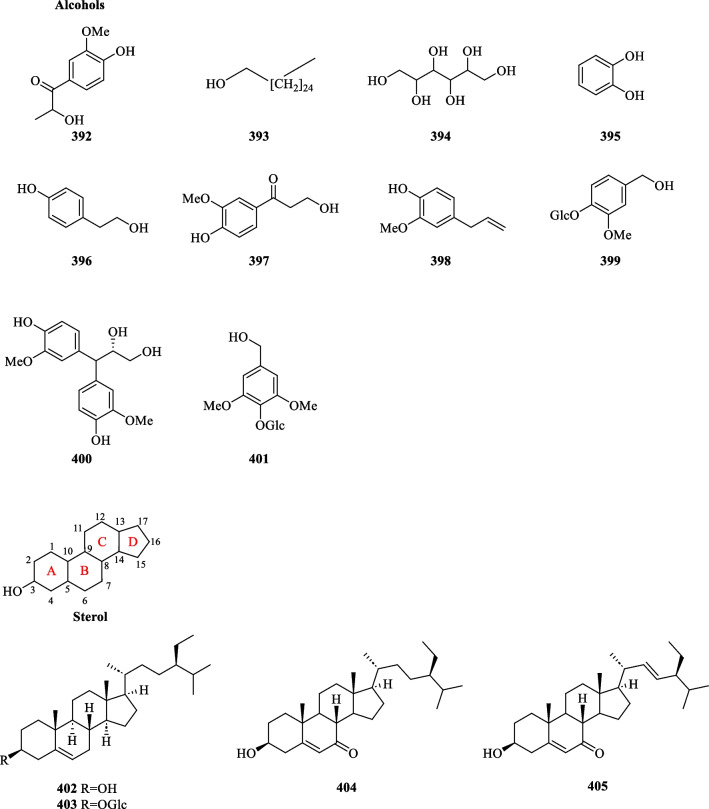
Alcohols (**392**–**401**) and sterols (**402–405**)

A total of ten alcoholic compounds (**392**–**401**) were reported from the whole plants of *S. glabra*, including a saturated fatty alcohol (**393**), a sugar alcohol (**394**), and eight phenol derivatives (**392**, **395**–**401**) (Table 18, Fig. 22).

**Table 18:** Alcohols, sterols, and other compounds from *S. glabra*

Chemical constituents	Molecular formula	Source	Fraction	References
Alcohols				
392. Evofolin A	C_10_H_12_O_4_	*S. glabra,* whole plant	EtOH	[59]
393. Hexacosanol	C_26_H_54_O	*S. glabra,* whole plant	EtOH	[102]
394. Hexitol	C_6_H_14_O_6_			
395. 1,2-Benzenediol	C_6_H_6_O_2_	*S. glabra,* whole plant	EtOAc	[78]
396. Tyrosol	C_8_H_10_O_2_		BuOH	
397. β-Hydroxypropiovanillone	C_10_H_12_O_4_	*S. glabra,* whole plant and stems	H_2_O, EtOAc	[64, 123]
398. Caryophyllic acid	C_10_H_12_O_2_	*S. glabra,* whole plant	H_2_O, EtOH	[124, 126]
399. Vanilloloside	C_14_H_20_O_8_	*S. glabra,* whole plant	BuOH, EtOAc	[64, 78]
400. (2*S*)-3,3-Di-(4-hydroxy-3-methoxyphenyl)-propane-1,2-diol	C_17_H_20_O_6_	*S. glabra,* whole plant	H_2_O	[128]
401. 3,5-Dimethoxyl-4-hydroxybenzyl alcohol 4*-O*-β-D-glucoside	C_15_H_22_O_9_	*S. glabra,* whole plant	EtOAc	[64]
Sterols				
402. β-Sitosterol	C_29_H_50_O	*S. glabra, S. hainanensis,* whole plant	EtOH, PE	[53, 85, 103]
403. Daucosterol	C_35_H_60_O_6_	*S. glabra,* whole plant	EtOH	[85, 102]
404. 3β-Hydroxystigmast-5-en-7-one	C_29_H_48_O_2_	*S. glabra,* whole plant	EtOAc	[36]
405. 3β-Hydroxystigmast-5,22-dien-7-one	C_29_H_46_O_2_			
Others				
406. 4-Hydroxy-4,7-dimethyl-1-tetralone	C_13_H_16_O	*S. glabra,* whole plant	EtOAc	[36]
407. Glucose	C_6_H_12_O_6_	*S. glabra,* whole plant	EtOH	[102]
408. *N*-*trans*-Feruloyltyramine	C_18_H_19_NO_4_	*S. glabra,* whole plant	EtOH	[88]
409. Desmethoxyyangonin	C_14_H_12_O_3_	*S. glabra*	–	[106]
410. Hexadecane	C_16_H_34_			
411. Aniba dimer A	C_28_H_27_O_3_			
412. ( +)-Toussaintin C	C_17_H_17_NO_3_	*S. glabra* subsp. *brachystachys*, whole plant	EtOAc	[73]
413. (−)-Toussaintin C

Sterols are a subgroup of steroids distinguished by their hydroxyl group at C-3 of ring A [127]. All four stigmastane-types phytosterols (**402**–**405**) reported in the literature were isolated from the whole plants of *S. glabra*, except for compound **402**, whose occurrence was also reported from the subspecies *S. hainanensis.*

### Isolation of other compounds from *S. glabra*

**Fig. 23:**
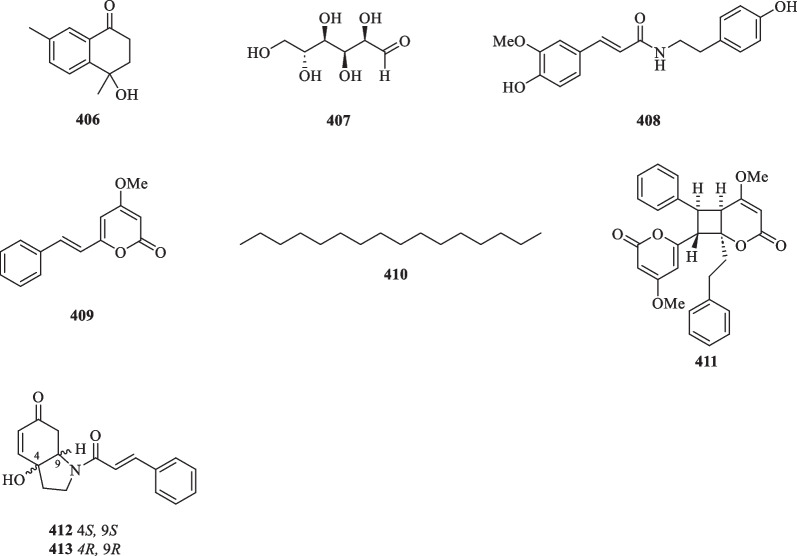
Other compounds (**406**–**413**)

Apart from the mentioned classes of secondary metabolites, the rare presence of a ketone (**406**), monosaccharide (**407**), alkaloids (**408**, **412–413**), alkane (**410**), and lactones (**409** and **411**) have also been reported during the isolation process (Table 18, Fig. 23).

## Biogenetic pathways of oligomeric sesquiterpenoids and meroterpenoids from *S. glabra*

The biosynthesis of oligomeric sesquiterpenoids and meroterpenoids from *S. glabra* is a complex process involving a series of reactions. Generally, the majority of lindenane-type oligomers in *S. glabra* were assembled via Diels–Alder reaction, facilitated by the presence of diene and dienophile elements within the monomeric units. Occasionally, the dimerisation or oligomerisation process also involves the integration of direct cyclisation, oxidative coupling, esterification, acetalisation, aldol reaction, and Michael-type reaction using various linkers [129]. Herein, the proposed biogenetic routes for a selection of oligomeric sesquiterpenoids and meroterpenoids isolated from *S. glabra* are summarised. A biogenetic relationship that connects the various key terpenoid skeletons obtained from *S. glabra* is also presented.

### Biosynthesis of shizukaol A and lindenatriene as a key biogenetic building block

**Scheme 1:**
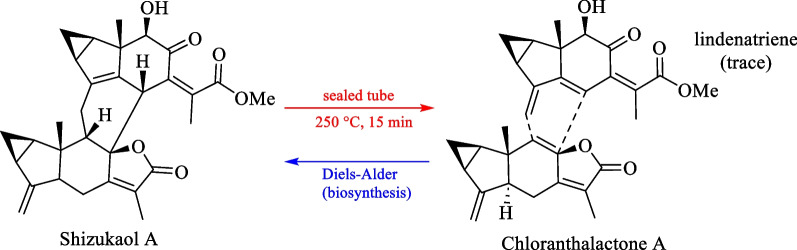
Pyrolysis of shizukaol A and the biosynthetic hypothesis of dimeric lindenanes [130]

The first dimeric lindenene sesquiterpene, shizukaol A, was reported by Kawabata et al. [130]. Since then, numerous other heterodimeric sesquiterpenes have been reported with a carbon skeleton similar to that of shizukaol A. Shizukaol A was hypothesised to be biogenetically derived from a Diels–Alder reaction (Scheme 1) based on a sealed tube pyrolysis experiment of shizukaol A, yielding lindenatriene and chloranthalactone A as the retro-Diels–Alder adducts [130]. However, since lindenatriene was highly unstable and rapidly decomposed in situ, it was only obtained in trace amounts and partially characterised by ^1^H NMR.

**Scheme 2:**
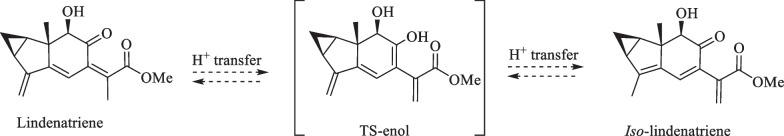
Proposed tautomerisation of lindenatriene and *iso*-lindenatriene

The structure proposed for lindenatriene by Kawabata’s group was finally confirmed almost two decades later through a synthesis by Eagan et al. [131]. They showed that lindenatriene was unstable and readily isomerised to its more thermodynamically stable tautomer, *iso*-lindenatriene, under mildly basic conditions (Scheme 2). Due to the inherent instability of the triene moiety, Yuan et al. [132] encountered a setback in obtaining the anticipated Diels–Alder adduct in their model biomimetic reaction, in which they employed the equally unstable *des*-hydroxy derivative of lindenatriene. Despite synthesising lindenatriene, the isolated and characterised compound was its tautomer, *iso*-lindenatriene. Consequently, the discrepancies between the ^1^H NMR chemical shifts reported by Yuan et al. and those reported by Kawabata et al. raised questions regarding the validity of the proposed biogenetic mechanism. Finally, Martinez et al. [133] re-assessed the literature and NMR data, affirming lindenatriene’s continued relevance as a building block in Kawabata’s original biogenetic hypothesis.

### Biosynthesis of sarglanoids A-C (28–30)

**Scheme 3:**
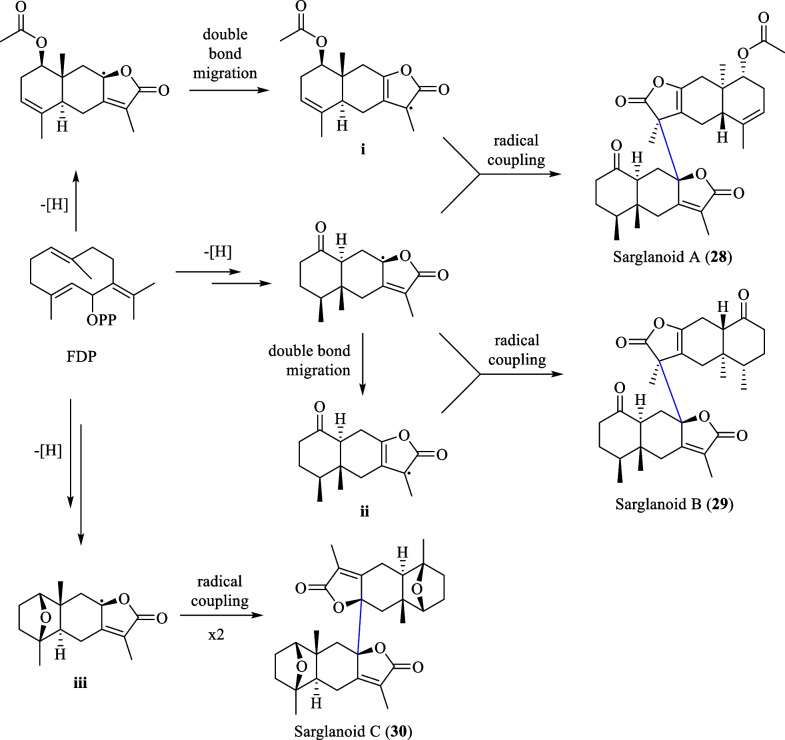
The biosynthetic route of compounds **28**–**30**

Li et al. [24] proposed that sarglanoids A-C (**28**–**30**) originate from a common precursor farnesyl diphosphate (FDP). A cascade of spontaneous cyclisation, oxidation, lactonisation, and double-bond migration was hypothesised to result in the formation of the eudesmane (**i**) and eremophilane (**ii**, **iii**) monomers (Scheme 3). Subsequently, the lactone moieties of the monomeric units are directly connected by a C–C bond via free-radical coupling reactions to form sarglanoids A-C.

### Biosynthesis of sarcanolides A-D (74, 75, 128 and 129)

**Scheme 4:**
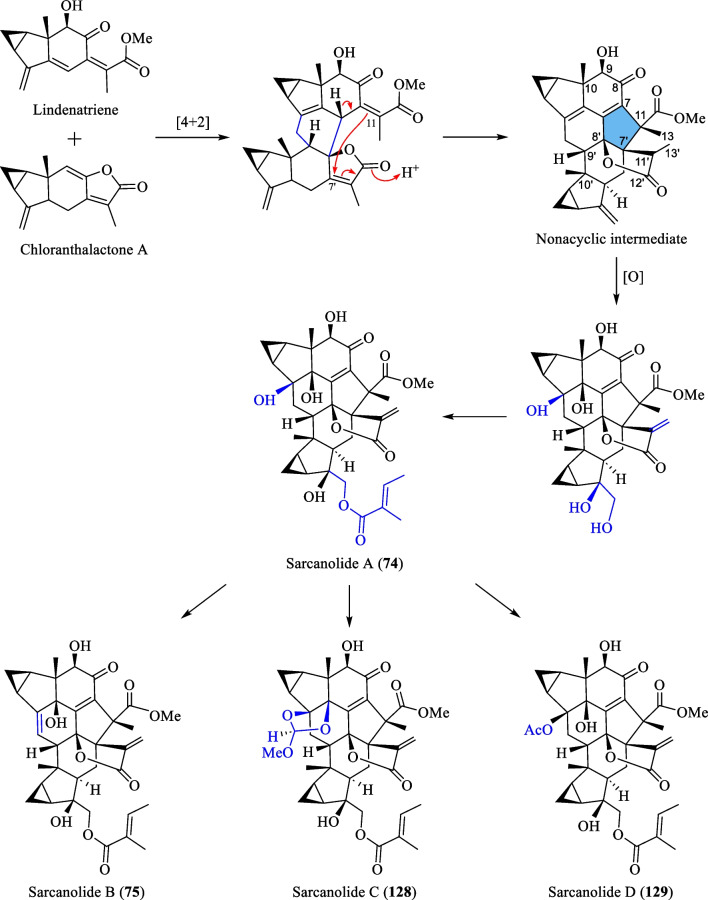
The proposed biosynthetic route of compounds **74**, **75**, **128** and **129**

As depicted in Scheme 4, He et al. [47] proposed a biosynthetic route for sarcanolides A (**74**), B (**75**), C (**128**) and D (**129**), which involves a Diels–Alder reaction between lindenatriene and chloranthalactone A. The resulting cycloadduct then undergoes an acid-catalysed intramolecular cyclisation connecting C-11 to C-7′, generating a β-oriented lactone. Sequential oxidation and acylation of the nonacyclic intermediate would ultimately form sarcanolide A (**74**), which could then be transformed into sarcanolides B, C and D (**75**, **128** and **129**) by dehydration, ortho-ester formation, and acetylation, respectively.

### Biosynthesis of sarglaperoxides A and B (98 and 99), sarcaglarols A-D (117–120), sarcaglarone A (141), and 6α-hydroxysarglaperoxide A (142)

**Scheme 5:**
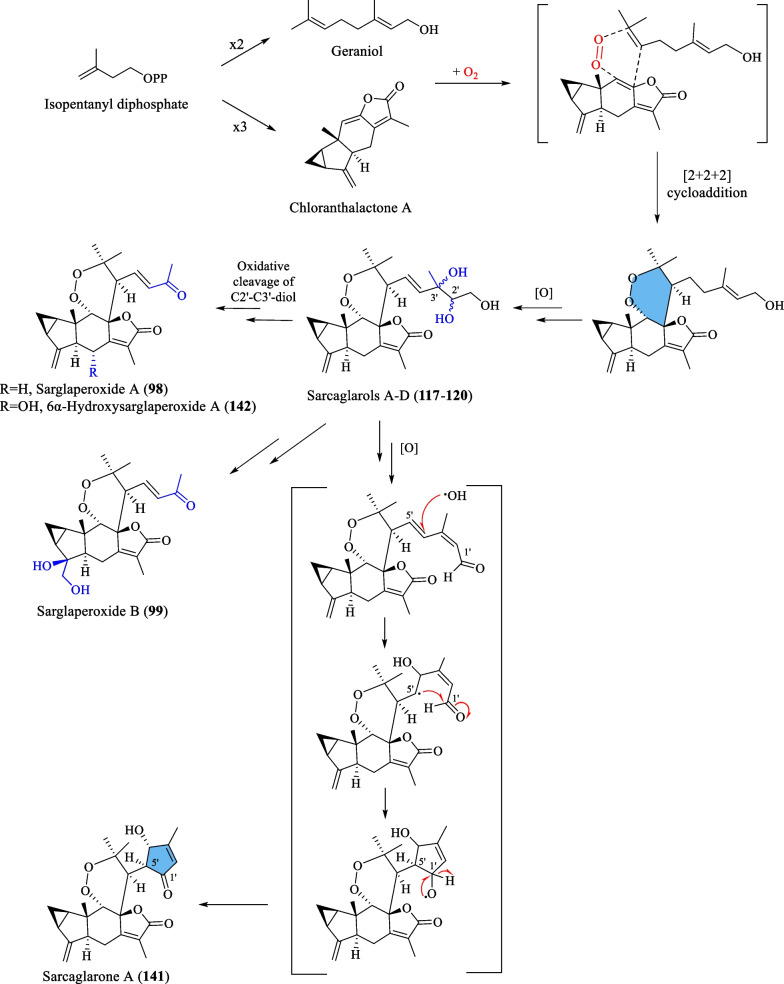
The proposed biosynthetic route of compounds **98**–**99**, **117**–**120**, **141** and **142**

Wang et al. [39] proposed a common biosynthetic pathway for compounds **98**–**99**, **117**–**120**, **141** and **142** as shown in Scheme 5. It was inferred that geraniol and chloranthalactone A, two abundant components of *S. glabra,* serve as biogenetic precursors for the formation of these dimers. The fundamental core of these dimers was hypothesised to be formed through a photocatalytic aerobic [2 + 2 + 2] cycloaddition step. Firstly, intermolecular cycloaddition between geraniol and chloranthalactone A in the presence of O_2_ generates an intermediate that yields sarcaglarols A-D (**117**–**120**) via a series of oxidative reactions. The sarcaglarols undergo successive oxidative cleavage at the C-2′,C-3′-diol fragment, leading to the formation of sarglaperoxide A (**98**) and its hydroxylated analogues, **99** and **142**. Finally, the formation of the lactone functionality in sarcaglarone A (**141**) involves oxidation of the sarcaglarols, followed by a free-radical-mediated intramolecular cyclisation that links C-1′ and C-5′.

### Biosynthesis of sarglalactones A-H (101–108)

**Scheme 6:**
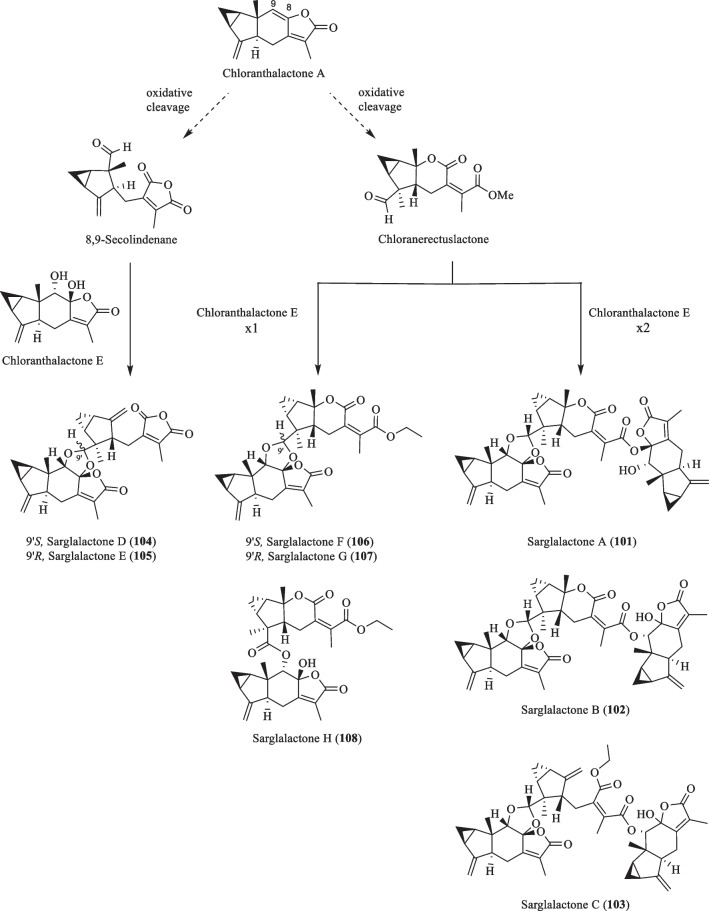
The proposed biosynthetic route of compounds **101**–**108**

Based on the postulation by Chi et al. [33], the oligomers sarglalactones A-H (**101**–**108**) could originate from chloranthalactone A, whose highly conjugated lactone undergoes oxidative cleavage at the Δ^8,9^ double bond to yield two presumptive precursors chloranerectuslactone and 8,9-secolindenane (Scheme 6). The skeleton of the trimers **101**–**103** arises from the condensation between a chloranerectuslactone core and two units of chloranthalactone E through acetalisation and transesterification, while the dimers **106**–**108** are assembled in the same manner, except that they lack a second unit of chloranthalactone E. On the other hand, epimers **104** and **105** are linked by the monomers, 8,9-secolindenane and chloranthalactone E, through a cyclic acetal moiety.

### Biosynthesis of sarcaglabrin A (109) and 7*′*-oxysarcaglabrin A (143)

**Scheme 7:**
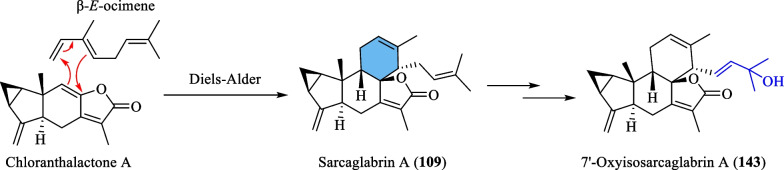
The proposed biosynthetic route of compounds **109** and **143**

Sarcaglabrin A (**109**) was presumed to be the Diels–Alder adduct of chloranthalactone A and a naturally occurring geranyl diphosphate (GDP)-derived monoterpene, β-*E*-ocimene (C_10_) [4]. On the other hand, the hydroxylated analogue **143** isolated by Sun et al. [40] was deduced to adopt a similar biosynthetic route (Scheme 7).

### Biosynthesis of sarglaromatics A-E (123–127)

**Scheme 8:**
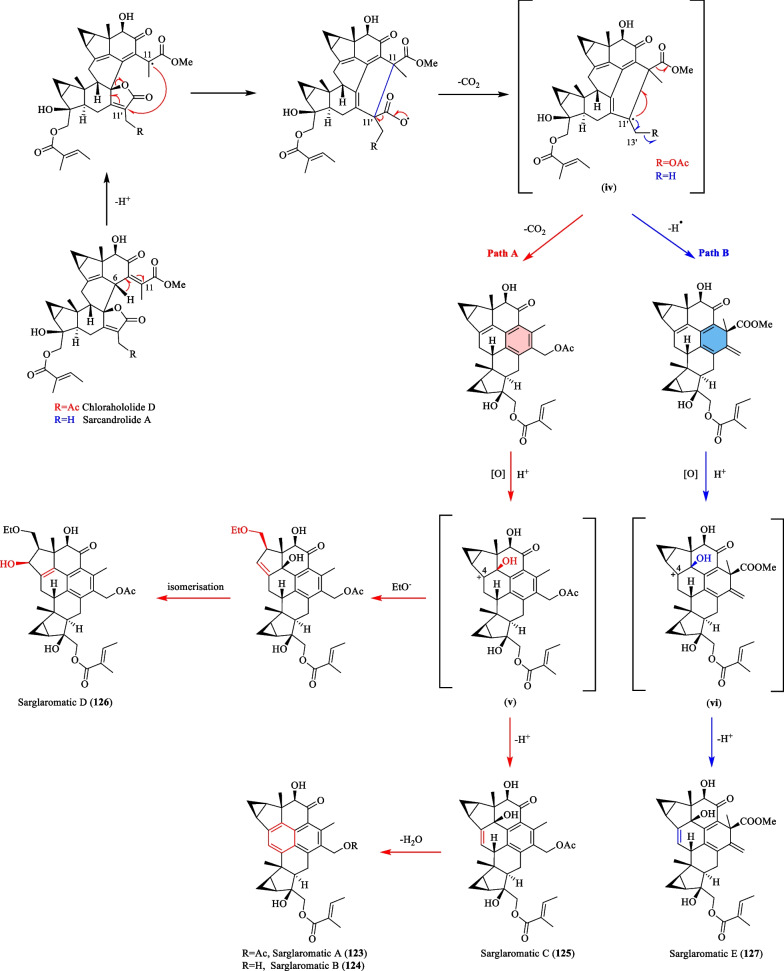
The proposed biosynthetic route of compounds **123**–**127**

According to Sun et al. [41], the unprecedented skeletons of sarglaromatics A-E (**123**–**127**) were presumed to derive from chlorahololide D (**80**) and sarcandrolide A (**69**), two abundant [4 + 2] lindenane sesquiterpenoid dimers from *S. glabra* (Scheme 8). The removal of H-6 of the precursors consequently generates a C-11 free radical that performs an intramolecular attack onto C-11′ of the lactone ring, thereby forming the key radical intermediate (**iv**). Subsequently, radical termination could occur in two possible ways. The first pathway (A) involves a decarboxylation process that gives rise to a dimer bearing an aromatic framework, which eventually transforms into sarglaromatics A-C (**123**–**125**) by way of a carbocation intermediate (**v**). Alternatively, the opening of the cyclopropane ring in the carbocation intermediate leads to the formation of sarglaromatic D (**126**). In the second pathway (B), the free radical at C-11′ is terminated with the formation of an exocyclic bond. By way of a carbocation formation at C-4 (**vi**), the corresponding dimer, sarglaromatic E (**127**), is formed.

### Biosynthesis of sarglafuran A (131)

**Scheme 9:**
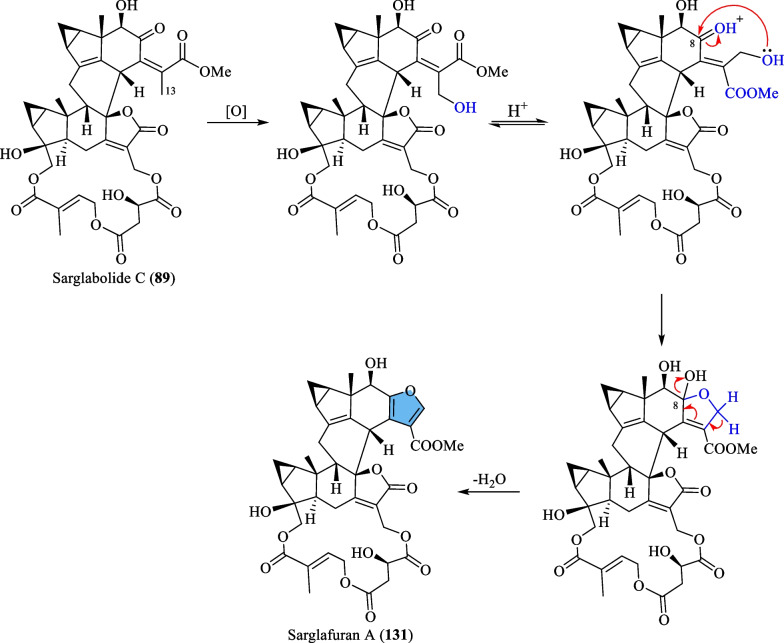
The proposed biosynthetic route of compound **131**

According to Wang et al. [43], sarglafuran A (**131**) was proposed to be biosynthesised through the hydroxylation of the C-13 methyl group in sarglabolide C (**89**), followed by an intramolecular nucleophilic addition to form a hemiketal intermediate. Finally, dehydration at C-8 furnishes the characteristic furan moiety in **131** (Scheme 9).

### Biosynthesis of sarglaoxolanes A-C (144–146)

**Scheme 10:**
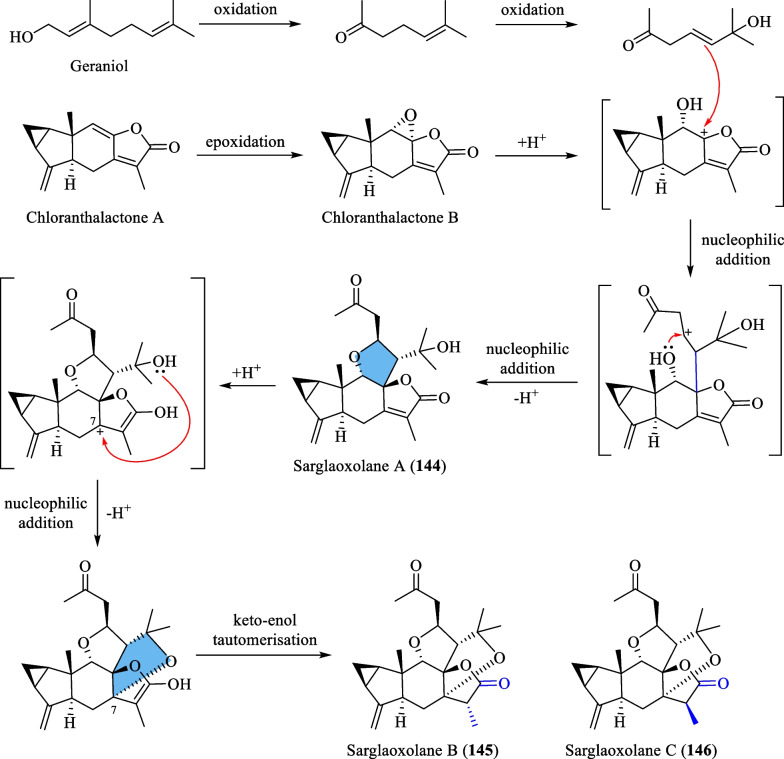
The proposed biosynthetic route of compounds **144**–**146**

A possible biogenetic and transformational pathway of sarglaoxolanes A-C (**144**–**146**) is presented in Scheme 10 [45]. The biosynthesis commences with the epoxidation and oxidation of the precursors chloranthalactone A and geraniol, which results in a carbocation intermediate and a tertiary alcohol, respectively. Subsequently, the skeleton of heterodimer **144** is constructed through two consecutive nucleophilic additions. Meanwhile, epimers **145** and **146** could be spontaneously formed from **144** by nucleophilic addition of the tertiary alcohol to the carbocation C-7, followed by tautomerisation.

### Biosynthesis of glabralides A-F (216–221)

**Scheme 11:**
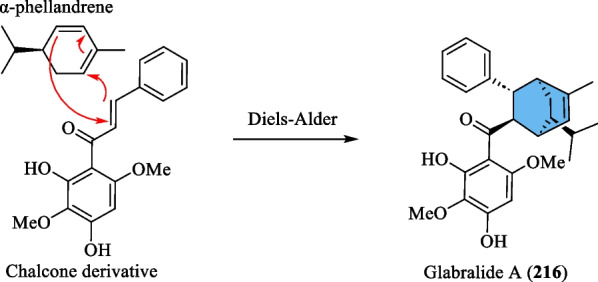
The biosynthetic route of compound **216**

**Scheme 12:**
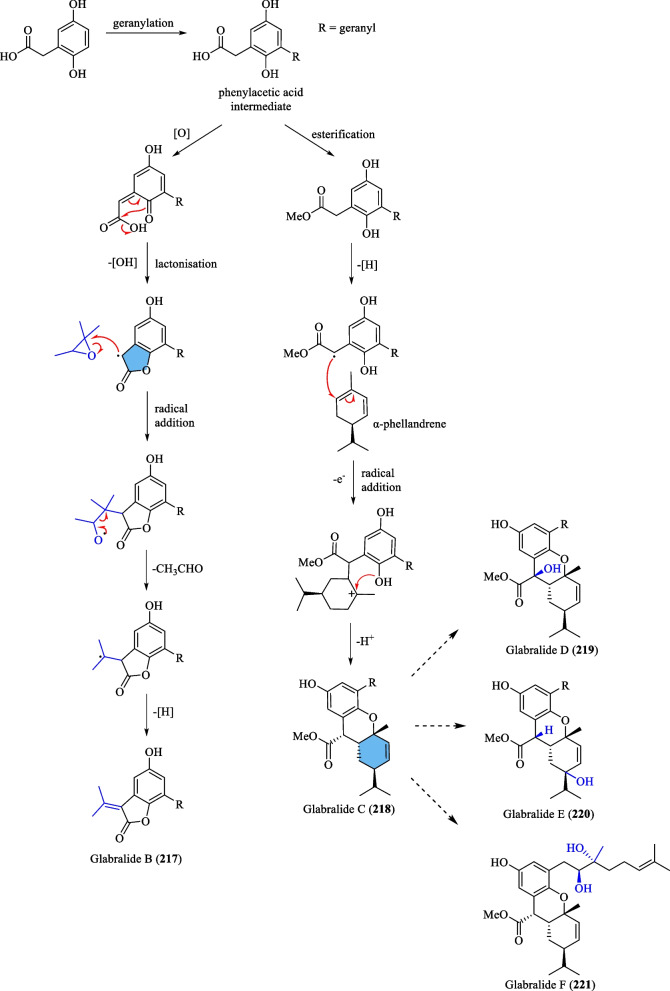
The biosynthetic route of compounds **217–221**

Yang et al. [72] proposed that the scaffold of glabralide A (**216**) is constructed via an enzyme-catalysed Diels–Alder reaction between α-phellandrene and a chalcone derivative (Scheme 11). On the other hand, glabralides B-F (**217**–**221**) were postulated to adopt a divergent route featuring a geranylated phenylacetic intermediate (Scheme 12). Oxidation of the phenylacetic intermediate forms a conjugated ketone moiety, which then undergoes radical lactonisation to give a lactone radical. Successive radical addition involving 2,2,3-trimethyloxirane, followed by an acetaldehyde elimination step eventually gives glabralide B (**217**). From the same phenylacetic intermediate, an alternative radical intermediate is formed through methyl esterification and oxidation, which, by radical addition and oxidation produces a tertiary carbocation intermediate. Subsequent cationic cyclisation and oxidation then give rise to a fused 6/6/6 ring system exhibited by glabralide C (**218**). Ultimately, hydroxylation of the ring system or the geranyl moiety would result in the formation of glabralides D-F (**219**–**221**).

### Biosynthesis of sarglamides A-E (224–228)

**Scheme 13:**
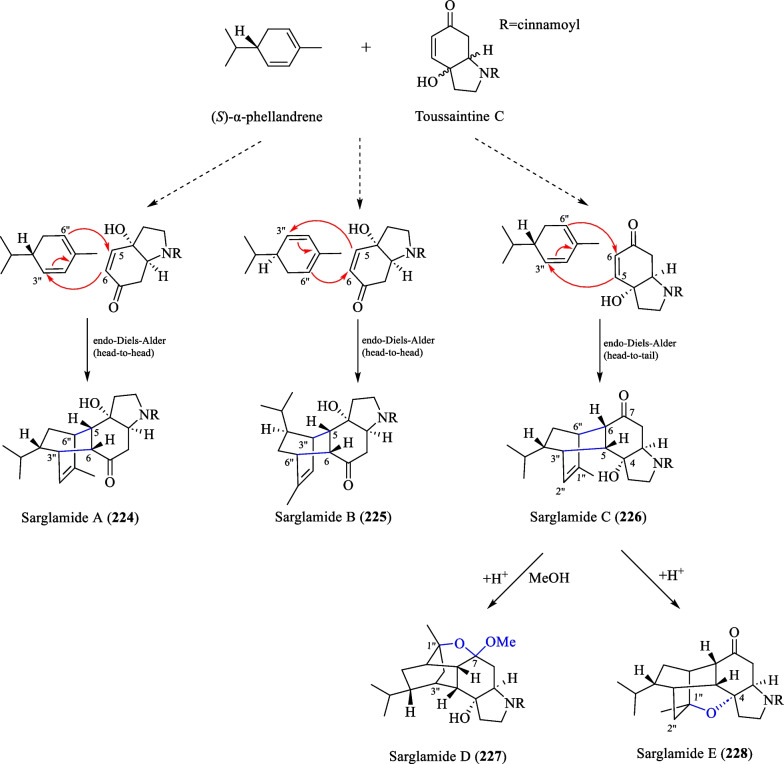
The biosynthetic route of compounds **224–228**

The indolidinoid-monoterpene conjugates, sarglamides A-E (**224**–**228**), were proposed to derive from the precursors (*S*)-α-phellandrene and toussaintine C (Scheme 13) [73]. The biosynthesis of sarglamides A and B (**224** and **225**) was assumed to involve a head-to-head *endo*-Diels Alder between (*S*)-α-phelladrene and (+)-toussaintine C. The two regioisomers differ in the approach direction of the (*S*)-α-phellandrene dieneophile during the reaction, with the isopropyl group assuming an α orientation for **224** and a β orientation for **225**. Conversely, sarglamide C (**226**) was postulated to form via an *endo*-Diels Alder between (*S*)-α-phellandrene and (-)-toussaintine C in a head-to-tail manner. Sarglamide D (**227**) could be derived from **226** via acetalisation at the C-7 carbonyl, followed by an electrophilic addition that links the C-7 oxygen to C-1″. On the other hand, an acid-catalysed electrophilic addition of the C-4 hydroxyl to the Δ^1′′,2′′^ alkene in **226** would give rise to **228**.

### Biogenetic relationship among various terpenoid skeletons of *S. glabra*

**Scheme 14:**
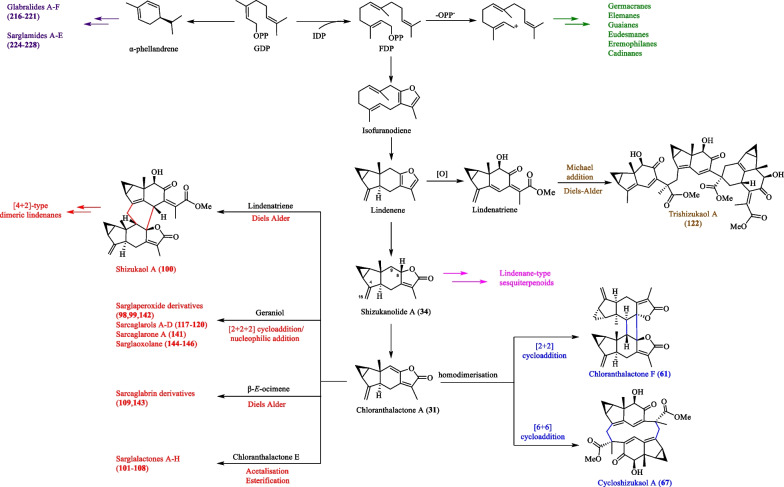
The biogenetic relationship among various terpenoid skeletons of *S. glabra*

A biogenetic pathway linking the various terpenoid skeletons of *S. glabra* is depicted in Scheme 14. The terpenoid constituents of *S. glabra* are produced via a common mevalonate pathway. Within this pathway, geranyl diphosphate (GDP) and farnesyl diphosphate (FDP) serve as pivotal intermediates, directing the synthesis of monoterpenoids and sesquiterpenoids, respectively. Cyclisation of GDP yields the monoterpene α-phellandrene, a precursor of meroterpenoids from *S. glabra*, e.g., glabralides A-F (**216**–**221**) and sarglamides A-E (**224**–**228**).

On the other hand, the addition of an isopentenyl diphosphate (IDP) unit to GDP forms FDP, an acyclic precursor of different classes of cyclic sesquiterpenes. The process involves the formation of the highly reactive farnesyl carbocation intermediate, which undergoes cyclisation and rearrangements to construct the diverse ring systems exhibited by germacrane, elemane, guaiane, eudesmane, eremophilane and cadinene-type sesquiterpenoids [134].

Alternatively, lindenane-type sesquiterpenoids may be synthesised from FDP through isofuranodiene, a naturally occurring constituent discovered in plants of the Chloranthaceae family [135]. Intramolecular cyclopropanation of isofuranodiene would generate lindenene, the biosynthetic precursor from which shizukanolide A and lindenatriene are derived. Through Michael addition and Diels–Alder reaction, lindenatriene could oligomerise into the trimer trishizukaol A (**122**), whereas modifications and functionalisation of shizukanolide A (**34**) at C-4, C-8, C-9, and C-15 enables its conversion into various lindenane-type sesquiterpenoids, including chloranthalactone A (**31**).

Chloranthalactone A (**31**) is a lindenane-type sesquiterpenoid with a highly unsaturated skeleton that exhibits versatility in undergoing dimerisation or oligomerisation via diverse pathways. The resulting array of dimeric and oligomeric sesquiterpenoids varies based on the number and positions of double bonds engaged in the reaction. For example, [2 + 2] and [6 + 6] cycloadditions between two units of chloranthalactone A (**31**) lead to the formation of the homodimers chloranthalactone F (**61**) and cycloshizukaol A (**67**), respectively.

Additionally, chloranthalactone A assumes a pivotal role as a building block in the biosynthesis of the [4 + 2]-type dimeric lindenanes, the chemotaxonomic constituents of *S. glabra*. The basic skeleton of this class of compounds is depicted by shizukaol A (**100**), a cycloadduct of chloranthalactone A and lindenatriene. Through subsequent transformations such as acetylation, esterification, oxidation, glycosylation, epoxidation, and lactonisation, shizukaol A can be transformed into various [4 + 2]-type congeners. Chloranthalactone A is also the putative precursor of a series of sesquiterpene-normonoterpene heterodimers, exemplified by compounds **98**, **99**, **141**, **142**, **117**–**120**, and **144**–**146**. The biosynthetic pathway occurs through [2 + 2 + 2] cycloaddition or nucleophilic addition reactions with geraniol. On the other hand, the conjugation of chloranthalactone A with a different monoterpene moiety, β-*E*-ocimene, would yield compounds **109** and **143**. The conjugation of chloranthalactone A derivatives with chloranthalactone E through an atypical acetalisation and esterification process would result in the formation of 8,9-secolindenane-derived dimers and oligomers (**101**–**108**).

## Biological activities

The multifarious biological activities of *S. glabra* revealed by modern pharmacological studies include antioxidative, antibacterial, antifungal, antiviral, antimalarial, anti-thrombocytopenic, antitumour, anti-inflammatory, immunomodulatory, hepatoprotective effects, etc. A compilation of the reported bioactivities exhibited by plant extracts, medicinal formulations, and isolates from *S. glabra* can be found in Table 19.

**Table 19:** Biological activities of *S. glabra*

Biological activity	Active fraction/compound	Study model	References
Antioxidative	SGE	Hydroxy radical (in vitro) & γ-ray-irradiated guinea pigs (in vivo)	[136]
	SGE	γ-ray-irradiated guinea pigs	[137]
	Aqueous extract	DPPH (in vitro)	[125]
	Caffeoylquinic acid, caffeic acid, isofraxidin (**230**), & rosmarinic acid 4-*O*-β-D-glucoside (**382**)	Restrained stressed mice	[139]
	CADPE	DPPH (in vitro)	[146]
	*S. glabra* powder	γ-ray-irradiated miniature pigs	[140]
	Flavonoids	DPPH, superoxide anion & hydroxy radical (in vitro)	[145]
	SGP-1	Hydroxy radical, superoxide anion, DPPH, ABTS & FRAP (in vitro)	[144]
	Astilbin (**335**) & rosmarinic acid (**369**)	Fenton-treated mesenchymal stem cells	[147]
	Phenolics, flavonoids & polysterol	DPPH (in vitro)	[141]
	EtOAc fraction	Scopolamine-induced cognitive deficit mice	[142]
	Rosmarinic acid (**369**)	γ-ray-irradiated rats	[118]
	*S. glabra*	X-ray- irradiated rats	[138]
	Proteoglycans	DPPH (in vitro)	[148]
	EtOH extract	DPPH, ABTS & FRAP (in vitro)	[143]
Antibacterial & Antifungal	Kaempferol 3-*O*-β-D-glucuronide (**310**)	*Streptococcus mutans*	[87]
	EtOAc & n-BuOH fractions	*Staphylococcus aureus*	[149]
	EtOH extract	*Propionibacterium acnes, Staphylococcus aureus*	[151]
	Sarglaperoxide A (**98**)	*Staphylococcus aureus*	[38]
	EtOAc & n-BuOH fractions	*Fusarium graminearum, Melanconium magnum, Alternaria alternate, Fusarium oxysporum, Trichophyton rubrum, Escherichia coli & Staphylococcus aureus*	[150]
	EtOH extract	*Colletotrichum gloeosporioides, Botryodiplodia theobromae & Phomopsis mangiferae*	[152]
Antiviral	SGE	Influenza-induced pneumonia in mice	[153]
	Rosmarinic acid 4-*O*-β-D-glucoside (**382**)	A/FM/1/47-H1N1-virus-infected mice	[154]
	Eleutheroside B_1_ (**232**)	RNP virus	[155]
	EtOH fraction	H1N1 virus-infected mice	[156]
	Isofraxidin (**230**)	Influenza A virus (IAV)-induced ALI in mice	[80]
	SGE	Influenza A H1N1	[157]
	SGE	HIV-1 PR & cathepsin-L PR	[158]
Antimalarial	13′-*O*-Methylsuccinylshizukaol C (**136**)	*Plasmodium falciparum*	[44]
Anti-thrombocytopenic	SGE	Babl/c mice	[159]
	Flavonoids	Cytarabine-induced thrombocytopenia in mice	[160]
	Flavonoids	Bone marrow failure mouse model	[161]
	Flavonoids	Bone marrow failure mouse model	[93]
	Flavonoids	Cytarabine-induced thrombocytopenia in mice	[162]
	Flavonoids	Rat bone marrow stromal cells & megakaryocytes	[94]
	Isofraxidin (**230**)	Arachidonic acid-induced platelet aggregation in mice	[80]
Antitumour	3,3′-Biisofraxidin (**233**), pinostrobin, isofraxidin (**230**), palmitic acid, atractylenolide III, chloranthalactone E (**33**), *N*-pentadecanoic acid (**354**), istanbulin A (**152**), uvangoletin (**272**) & scoparone (**234**)	HL-60 cells	[85]
	EtOAc fraction	HL-60 cells	[164]
	DCM fraction	U937 cells	[165]
	*Zhongjiefeng* injection	Bel 7402 & HCT-8 cells	[170]
	SGE	Mice CNE1 & CNE2 cells	[166]
	*Zhongjiefeng* injection	A-549, HCT-29 & BGC-823 cells	[171]
	*Zhongjiefeng* injection	SGC-7901 mice tumour	[172]
	Sarcandrolides A-C (**69**–**71**)	HL-60 & A-549 cells	[17]
	CADPE	Human cancer cells	[146]
	Sarcandracoumarin (**244**)	HeLa & A549 cells	[78]
	CADPE	AGS, HGC27, H1299, A549, HCT116 p53 WT & U2OS cells	[178]
	Eleutheroside B_1_ (**232**)	BGC-823 & A2780 cells	[79]
	*Zhongjiefeng*	DU-145 cells	[173]
	Sarcandrolides F & H (**81** & **83**)	HL-60 cells	[22]
	CADPE	Mice H22 & S180 cells	[179]
	EtOH extract	HL-60 & HT-29 cells	[167]
	SGP-2	MG-63 & S-180 cells	[180]
	SGP-2	MG-63 cells	[181]
	3,3′-Biisofraxidin (**233**)	BGC-823 cells	[174]
	Uvangoletin (**272**)	HL-60 cells	[175]
	*S. glabra* solution	DU-145 cells	[168]
	Flavonoids	K562 cells	[176]
	Sargalactones A, D-H	U2OS cells	[33]
	Shizukaols C and D (**64** and **78**), chlorahololide D (**80**) & sarcandrolide E (**73**)	Hela & MCF7 cells	[4]
	Proteoglycans	U2OS & MG63 cells	[148]
	PE & EtOAc extracts (subsp. *hainanensis*)	BEL-7402, A549, HECT-8 & T-47D cells	[169]
	CADPE	CCRF-CEM, MOLT-4, HL-60 & K-562 cells	[182]
	Uvangoletin (**272**)	HepG2 cells	[177]
Anti-inflammatory	EtOAc extract & polysaccharides	LPS-induced RAW264.7 macrophage	[183]
	SGE	Restraint-stressed mice	[187]
	Isofraxidin (**230**)	LPS-induced inflammation in mice	[188]
	Isofraxidin (**230**)	LPS-induced ALI in mice	[189]
	Sarglabolide A (**87**), shizukaol G & shizukaol B	LPS-induced RAW264.7 macrophage	[37]
	SGE	LPS-induced ALI in mice	[190]
	*Zhongjiefeng* injection	LPS-induced ALI in mice	[191]
	Sarglaperoxide A (**98**)	LPS-induced RAW264.7 macrophage	[38]
	Sarglabolide L (**52**)	LPS-induced RAW264.7 macrophage	[23]
	Chloranthalactone B (**32**)	LPS-induced RAW264.7 macrophage	[184]
	3,5-Dihydroxycoumarin-7-*O*-α-L-rhamnopyranosyl-2H-chromen-2-one (**247**)	LPS-induced RAW264.7 macrophage	[82]
	Methyl isorinate	LPS-induced RAW264.7 macrophage	[185]
	Shizukaol D (**78**)	LPS-induced RAW264.7 macrophage	[48]
	Astilbin (**335**)	Human osteoarthritis chondrocytes & mouse OA model	[192]
	Chlorogenic acid (**377**), isofraxidin (**230**), & rosmarinic acid (**369**)	LPS-induced ALI in mice	[3]
	Shizukaol A (**100**)	LPS-induced RAW264.7 macrophage	[186]
	Sarcanolides C-E (**128**–**130**)	LPS-induced RAW264.7 macrophage	[42]
	Sarglanoid C (**25**)	LPS-induced RAW264.7 macrophage	[25]
	Sarglanoid C (**30**), linderaggredin D (**21**)	LPS-induced RAW264.7 macrophage	[24]
	Glabralides G and H (**222** and **223**) & sarglabolides B and C (**88** and **89**)	LPS-induced BV2 cells	[49]
	SERP 30	LPS-induced ARD in mice	[193]
	Trishizukaol A (**122**)	LPS-induced RAW264.7 macrophage	[50]
	Polysaccharides	Exercise-induced muscle damage in rats	[194]
	Sarglamides C-E (**226**–**228**) & toussaintine C	LPS-induced BV-2 microglial cells	[73]
Immunomodulatory	SGE	Restraint-stressed mice	[2]
	SGE	Restraint-stressed mice	[139]
	Polysaccharides	RAW264.7 macrophage cells	[195]
	p-SGP	B16F10 & CT26 cells (in vitro) & immunised mice (in vivo)	[196]
Hepatoprotective	Sarcaglabosides A-E (**2**, **3**, **165**, **166** and **148**) & chloranoside A (**35**)	D-galactosamine-induced toxicity in WB-F344 cells	[14]
	SGE	*Propionibacterium acnes &* LPS-induced immunological hepatitis in mice	[197]
Gastroprotective	SGE	Ethanol-induced gastric ulcer in rats	[198]
Hypoglycaemic	SGP-2	Streptozotocin-induced diabetes in mice	[199]
	SERP1	High-fat diet & streptozotocin -induced diabetes in mice	[200]
	CMSERP	pNPG assay (in vitro)	[201]
Hypolipidemic	Flavonoids	High-fat diet & streptozotocin -induced diabetes in mice	[202]
	Sarcaglarol A (**117**)	L02 cells	[39]
	Sarglaromatics A & B (**123** & **124**)	L02 cells	[41]
Anti-multidrug resistance	Sarglalactones A-H (**101**–**108**)	MCF-7/DOX cells	[33]
Autophagy-inducing activity	Glabratins A (**279**), D-F (**282**–**284**), I-N (**287**–**289** and **337**–**339**) & adunctin E (**293**)	HEK293 cells	[98]
Neuroprotective	5-Methoxy-6,7-methylenedioxycoumarin (**252**)	Enzymology assay	[83]

### Antioxidative

The antioxidative properties of *S. glabra* extract (SGE) have been widely investigated in several animal models. Preliminary and in vivo studies reported that SGE can ameliorate radiation-induced reactive oxygen species (ROS) injury and aid post-radiation recovery in guinea pig and rat models [136–138], besides moderating stress-attenuated immune response in restrained mice [139]. In miniature pigs, *S. glabra* powder exhibited prominent scavenging activities against radiation-induced ROS in the parotid gland [140].

The antioxidant properties of SGE exhibited concentration-dependent behaviour, as demonstrated by in vitro free radical scavenging assays [141, 142]. The most pronounced antioxidant efficacy was observed in the 75% ethanolic stem extract and 95% ethanolic leaf extract as determined by a comprehensive assessment employing 2,2-diphenyl-1-picrylhydrazyl (DPPH), 2,2′-azino-bis(3-ethylbenzthiazoline-6-sulfonic acid) (ABTS), and ferric reducing antioxidant power (FRAP) assays [143]. The antioxidative potential of *S. glabra* could be ascribable to the presence of phytochemical constituents such as phenolic acids, flavonoids, and polysaccharides, which possess intrinsic redox properties that help stabilise ROS due to their structural conformations [125, 144–146]. For example, the primary polyphenols and flavonoids of *S. glabra*, namely rosmarinic acid (**369**) and astilbin (**335**), possess the ability to scavenge ROS via hydrogen/electron transfer or Fe^2+^ chelation [147]. Rosmarinic acid (**369**) was also found to attenuate ROS by regulating PGC1-a/NOX4 signalling [118].

It was also established that the phenolic-rich ethyl acetate fraction of SGE manifested a strong antioxidative activity (IC_50_ = 6.84 ± 0.45 µg/mL) and displayed neuroprotective effects by enhancing cholinergic signalling in cognitive deficit mice [142]. In a bioactive structural basis study, it was unveiled that the antioxidant efficacy of proteoglycans from *S. glabra* was attributed to the presence of monosaccharides, namely xylose, glucosamine, and glucuronic acid [148].

### Antibacterial, antifungal and antiviral

Preliminary studies on SGE have shown promising results regarding its antimicrobial activity, among which ethyl acetate and n-butanol fractions in particular exhibited excellent inhibition against pathogenic bacterial strains in the disk diffusion test [87, 149]. The n-butanol extract of *S. glabra* was also recently reported to show minimum inhibitory concentrations of 15 mg/mL and 10 mg/mL against *Staphylococcus aureus* and *Escherichia coli,* respectively [150]. In another study, sarglaperoxide A (**98**), an isolate from the seeds of *S. glabra*, displayed 64.5% inhibition against *S. aureus* at a concentration of 25 μg/mL [38]. In addition to demonstrating bacteriostatic effects against *Propionibacterium acnes* [151], the ethanolic extract of *S. glabra* displayed notable antifungal activities against *Phomopsis mangiferae* at 2.5 mg/mL. Over a period of seven days post-treatment, the maximum inhibition rate reached a notable 50.29% [152].

The antiviral properties of SGE have also been well-characterised and extensively studied, particularly on influenza viruses [80, 153–157]. Rosmarinic acid 4-*O*-β-D-glucoside (**382**) was reported to mitigate pulmonary oedema and post-influenza infection by impeding virus proliferation [154], while eleutheroside B_1_ (**232**) displayed broad-spectrum antiviral activities in vitro, with IC_50_ ranging from 64 to 125 μg/mL [155]. Subsequent investigations into the structure–activity relationship of eleutheroside B_1_ (**232**) confirmed the significance of its aglycone moiety in mediating the observed viral nucleic acid suppression. Jin et al. [80] reported that isofraxidin (**230**) effectively inhibited platelet aggregation through the PI3K/AKT and mitogen‑activated protein kinase (MAPK) pathways, thereby alleviating lung inflammation induced by influenza A. In the work by Pan et al. [158], SGE was found to inhibit HIV-1 protease and cathepsin L with IC_50_ values ranging from 0.003 to 0.07 mg/mL and 0.11 to 0.26 mg/mL, respectively. Notably, chlorogenic acid (**377**), a prominent constituent of the extract, displayed the most potent inhibitory activity against the two viral proteases. The unique dual-inhibitory function of SGE and its active components against viral proteases could be useful in discovering lead compounds for developing new antiviral agents.

### Antimalarial

An assessment of antimalarial properties has been conducted on several compounds derived from *S. glabra* [44]. Among the compounds, 13′-*O*-methylsuccinylshizukaol C (**136**) exhibited remarkable efficacy against chloroquine-resistant *Plasmodium falciparum*, with an EC_50_ value of 4.3 pM. This potency surpassed that of artemisinin by approximately 1,000-fold. Furthermore, the compound demonstrated exceptional selectivity against the malaria parasite, as confirmed through a toxicity evaluation (IC_50_ = 39.0 ± 1.3 µM) conducted on a mammalian embryonic cell line.

### Anti-thrombocytopenic

The anti-thrombocytopenic properties of SGE have been extensively studied on mice models [80, 93, 94, 159–163]. It was unveiled that SGE promoted platelet activation and prevented platelet apoptosis via the mitochondrial pathway. Its effective constituents, specifically flavonoids, played a role in regulating mitochondrial transmembrane potential, externalisation of phosphatidylserine, and expressions of pro-apoptotic markers on circulating thrombocytes [93, 161]. At 63 mg/kg and 94.5 mg/kg, *S. glabra* flavonoids significantly increased the number of peripheral platelets and the polyploid ratio of megakaryocytes (p < 0.01) in thrombocytopenic mice [162]. The mechanism was associated with the elevation of thrombopoietin levels, which triggered megakaryocyte differentiation via the TPO-C-mpl pathway [162, 163].

### Antitumour

*S. glabra* has gained recognition for its significant cytotoxic effects. Extensive in vitro and in vivo investigations have been conducted over the past decades on various cell lines, focusing on plant extracts [164–169], medicinal formulations [170–173], and chemical constituents. The main cytotoxic chemical components identified from *S. glabra* include sesquiterpenoids [4, 17, 22, 33], coumarins [78, 79, 85, 174], flavonoids [175–177], and polysaccharides [146, 148, 178–182].

A number of isolates were reported to exhibit selective and potent activities against a panel of cell lines. For example, sarcandrolides A-C (**69**–**71**) significantly inhibited HL-60 leukocyte cell line with IC_50_ < 10 μM [17], whereas sarcandracoumarin (**244**) demonstrated moderate activity against human cervical carcinoma (HeLa) with an IC_50_ value of 49.3 μg/mL [78]. In a combination therapy, sarglalactones A-H (**101**–**108**) and doxorubicin exhibited exceptional synergistic cytotoxic effects on human osteosarcoma epithelial cells (U2OS) [33].

Caffeic acid 3,4-dihydroxyphenethylester (CADPE), a natural polyphenol derived from the aqueous extract of *S. glabra*, exhibits potent anticancer attributes. Its proposed mode of action involves the initiation of tumour senescence via the Twist1-mediated signalling pathway [178]. In a pharmacokinetic investigation, CADPE rapidly underwent hydrolysis into its anticancer metabolites, hydroxytyrosol and caffeic acid [179]. Additionally, CADPE regulated glycogen synthase kinase-3β (GSK3β), prompting the ubiquitin-dependent degradation of the proto-oncogene c-Myc [182]. Consequently, this modulates cell cycle regulators and anti-apoptotic proteins, resulting in tumour cell cycle arrest and apoptosis.

Malignant tumours frequently exhibit increased expression of eukaryotic initiation factor 4F (eIF4F), a protein primarily modulated by mitogen-activated protein kinase (MAPK). An acidic polysaccharide derived from *S. glabra* (SGP-2) employs this mechanism to trigger a MAPK-mediated intrinsic apoptosis pathway, effectively impeding tumour growth in both human and murine models [180]. Furthermore, the polysaccharide demonstrates excellent anti-proliferative effects on human osteosarcoma MG-63 cells, achieved through regulation of apoptotic cell population and activation of caspase-3 [181].

Validated through an in vivo xenograft assay, a preliminary study by Zheng et al. [175] validated the antitumour effects of uvangoletin (**272**) on HL60 cells. The proposed mechanism involves the interaction between mitochondria-mediated apoptotic proteins, leading to cytochrome C release into the cytosol and subsequent activation of apoptosis-executing caspases. Building upon this finding, Shen et al. [177] reported the promising cytotoxic activities of uvangoletin (**272**) on hepatocellular carcinoma cells (HepG2). This was evidenced by detected autophagy and apoptosis both in vivo and in vitro, coupled with metastasis suppression. The likely underlying mechanism involves the modulation of MAPK, AKT/mTOR, and TGF-β/smad2 signalling pathways.

### Anti-inflammatory

*S. glabra* is renowned for its remarkable anti-inflammatory activities, substantiated by multiple studies reporting its bioactivities in vitro [23–25, 37, 38, 42, 48–50, 73, 82, 183–186] and in vivo [3, 187–194]. The anti-inflammatory properties were found to be imparted by the presence of polysaccharides [183, 193, 194], phenolics [3, 185], coumarins [3, 82, 188, 189], and flavonoids [192].

Among the compounds identified from *S. glabra*, lindenane-type sesquiterpenoids exhibit prominent potential as anti-inflammatory agents, with extensive studies conducted on their effectiveness against lipopolysaccharide (LPS)-induced RAW264.7 macrophage [23–25, 37, 38, 42, 48–50, 73, 184, 186]. Three newly characterised dimeric lindenanes, namely sarcanolides C-E (**128**–**130**), showed greater inhibition of LPS-induced nitric oxide (NO) production compared to the positive control (L-NMMA) at a concentration of 25 μM. The observed IC_50_ values for these dimers ranged from 13.4 to 17.2 μM [42]. Additionally, sarglanoid C (**25**) was recently revealed to display anti-inflammatory effects on LPS-induced RAW 264.7 cells. The IC_50_ value recorded was 20.00 ± 1.30 μM, indicating a twofold potency compared to L-NMMA (IC_50_ = 41.40 ± 2.30 μM) [25].

In another study, Li et al. [24] reported the promising anti-inflammatory properties of linderaggredin D (**21**) and sarglanoid C (**30**). Their IC_50_ values were 25.7 ± 0.2 μM and 11.5 ± 0.3 μM, respectively, which were comparable to that of the positive control, dexamethasone (IC_50_ = 9.3 ± 0.2 μM). Bioinformatics and transcription factor analysis revealed that the anti-inflammatory activity of linderaggredin D (**21**) was associated with multiple pathways related to transcription factor NF-κB, a key component in inflammation progression.

A neuroinflammatory assay utilising the Griess reaction was employed to evaluate the anti-neuroinflammatory potential of meroterpenoids and sesquiterpenoid dimers derived from *S. glabra* [49]. These compounds exhibited significant inhibitory effects comparable to dexamethasone at concentrations < 5 µM. Utilising protein–protein interaction (PPI) network analysis and molecular docking, the anti-inflammatory mechanisms of the meroterpenoids glabralides G and H (**222** and **223**) were predicted. The PPI analysis indicated a prominent interaction with Hsp90AA1, a heat shock protein associated with neuroinflammation.

Regarding mechanistic action, Wei et al. [48] proposed that shizukaol D (**78**) elicits its anti-inflammatory effects by activating the AKT/Nrf2/HO-1 signalling cascade, which subsequently enhances the activity of antioxidant enzymes such as superoxide dismutase (SOD), glutathione (GSH), and glutathione peroxidase (GSH-px). Additionally, shizukaol A’s potent inhibitory effect on NO (IC_50_ = 13.79 ± 1.11 μM) was attributed to its capacity to trigger antioxidant genes and regulate oxidative stress via the HMGB1/Nrf2/HO-1 pathway [186].

The anti-inflammatory activities of a *S. glabra* polysaccharide, SERP-30, were reported for the first time by Feng et al. [193]. Corroborated by western blotting results, the postulated mechanism of SERP 30 involves the inhibition of LPS-induced phosphorylation of p38 and p65 via NF-κB and MAPK signalling pathways, leading to the protection of endothelial glycocalyx in LPS induced-acute respiratory distress syndrome (ARDS) in mice. In addition, the polysaccharides sourced from *S. glabra* exhibited the capacity to mitigate muscular injury in rats subjected to prolonged high-intensity exercise. This effect was achieved by elevating the levels of recuperative enzymes and cytokines such as creatine kinase (CK), lactate dehydrogenase (LDH), and tumour necrosis factor (TNF-α) [194].

The anti-neuroinflammatory activities of three indolidinoid-monoterpene compounds isolated from *S. glabra*, sarglamides C-E (**226**–**228**), along with toussaintine C were recently reported [73]. These compounds were evaluated for their impact on NO production in BV-2 microglial cells induced by LPS. Notably, sarglamides C-E (**226**–**228**) exhibited considerable inhibitory activities (> 30%) at concentrations < 20 µM, while showing no cytotoxicity on BV-2 cells (cell viability > 80%). On the other hand, the enantiomers of toussaintine C, namely (+)-toussaintine C (**412**) and (-)-toussaintine C (**413**), displayed similar effects at 5 µM.

### Immunomodulatory

As evidenced by multiple studies [2, 139], SGE was found to enhance immunity by mediating immune response and balancing the proportion of lymphocytes in restraint mice models. Comparable immunoprotective effects were also observed in *S. glabra*-derived polysaccharides, wherein increased expression of cell surface molecules and immune factors (IL-1β, IL-10, and iNOS) was observed in RAW 264.7 cells [195]. An acidic polysaccharide purified from *S. glabra* (p-SGP) was found to stimulate anti-tumour immune responses, making it an ideal candidate as a tumour vaccine adjuvant. The mechanism involves the upregulation of delta-like ligand 4 (DLL4) gene, which in turn activates the differentiation and maturation of T-helper cells and dendritic cells [196].

### Hepatoprotective

A group of sesquiterpene glycosides, namely sarcaglabosides A-E (**2**, **3**, **165**, **166** and **148**), together with chloranoside A (**35**) exhibited notable inhibitory activities in vitro at a concentration of 10^–4^ M [14]. Among them, sarcaglaboside B (**3**) exhibited the most pronounced hepatoprotective effect, displaying an inhibition rate of 65.9%. Furthermore, Li et al. [197] reported that SGE exerted hepatoprotective effects on mice models by inhibiting the activities of alanine aminotransferase level (ALT) and leukotriene B. The inhibitory rates were 78.5%, 70.3%, and 55.1% at concentrations of 500, 250, and 125 mg/kg, respectively.

### Gastroprotective

Based on a histopathological examination and conjoint metabolomics and network analysis, SGE was also found to associate with multiple signalling pathways related to gastric cell inflammation, metabolism, apoptosis, and differentiation [198]. The extract exerted profound protective effects on the gastric mucosa of rat models by alleviating oxidative stress, promoting antioxidant activity, and inhibiting the expression of inflammatory factors.

### Hypoglycaemic and hypolipidemic

It was reported that SGP-2 and SERP 1, two proteoglycans isolated from *S. glabra*, demonstrated remarkable α-glucosidase inhibitory activities, with IC_50_ values of 87.06 ± 11.76 μg/mL and 49.01 μg/mL, respectively [199, 200]. These polysaccharides mitigated insulin resistance, improved lipid metabolism, and attenuated oxidative stress under hyperglycaemic conditions through α-glucosidase inhibition. Similarly, CMSERP, a carboxymethylated polysaccharide from *S. glabra*, displayed substantial hypoglycaemic effects, achieving a maximum inhibition of 83.38% ± 2.30% at 1000 μg/mL concentration [201].

In addition to flavonoids [202], sesquiterpene dimers from *S. glabra* were discovered to possess notable hypolipidemic effects. Sarcaglarol A (**117**) reduced lipid droplets in L02 cells as indicated by oil red O staining, while sarglaromatics A and B (**123** and **124**) effectively mitigated lipid accumulation in L02 cells exposed to free fatty acids [39, 41]. The inhibitory potential of the dimers was postulated to be amplified by the free hydroxyl groups present in their structures, suggesting potential application in treating non-alcoholic steatohepatitis.

### Other biological activities

Apart from the mentioned biological activities, *S. glabra* and its chemical constituents were evaluated for their anti-multidrug resistance, neuroprotective, and autophagy-inducing activities. Chi et al. [33] described the anti-multidrug resistance against MCG-7/DOX cells displayed by several sesquiterpenoids isolated from the leaves of *S. glabra*, whose reversal fold values ranged from 11.8 to 129.2.

Du et al. [83] recently disclosed the acetylcholinesterase (AchE) inhibiting activity of coumarins from *S. glabra*. Notably, 5-methoxy-6,7-methylenedioxycoumarin (**252**) exhibited significant AchE inhibitory efficacy (IC_50_ = 1.982 ± 0.003 μM), surpassing donepezil (IC_50_ = 3.118 ± 0.006 μM). Molecular docking results showed that the main interaction involved hydrogen bonding with two target amino acids, Phe-288 and Arg-289. Additionally, Liu et al. [98] introduced the first report on the autophagy-inducing effects of *S. glabra* compounds, including glabratin A (**279**), glabratins D-F (**282**–**284**), glabratins I-N (**287**–**289** and **337**–**339**), and adunctin E (**293**). The compounds demonstrated enhanced conversion of the protein light chain LC3-II to its non-lipidated form (LC3-I) in vitro.

## Conclusions

This paper presents a comprehensive review on the compound isolation, biosynthesis, and pharmacological attributes of *S. glabra*. In essence, *S. glabra* is a highly prolific producer of secondary metabolites, including terpenoids, coumarins, lignans, flavonoids, sterols, anthraquinones, organic acids, and organic esters, several of which hold substantial research value due to their unique chemical structures and extensive range of biological effects. The taxonomical markers of this plant include lindenane-type sesquiterpenoids, characterised by a distinct linear 3/5/6 polycyclic ring and typically formed through a [4 + 2] Diels–Alder cycloaddition.

Through an analysis of existing literature, several gaps in research have been discerned. In the domains of phytochemistry and pharmacology, certain subspecies of *S. glabra* are underexplored. Continued research on these lesser-known variants holds the potential to unveil therapeutic properties or reveal hitherto unprecedented compounds. Despite the wide distribution of *S. glabra* across Asia, the majority of investigations have focused on specimens collected exclusively from China. Exploring specimens from diverse geographical origins would be of interest, facilitating a comprehensive understanding of common and distinct secondary metabolites among these plants.

From a biological activity viewpoint, *S. glabra* holds promise for further exploration of its pharmacological potential. Future studies are expected to analyse the mechanism of action of the active principles and evaluate the possible synergistic action within SGE. Besides, the establishment of precise analytical methods is crucial to standardise the secondary metabolites present in medicinal preparations like *ZhongJieFeng,* an herbal extract of *S. glabra* with excellent antitumour and anti-inflammatory activities*.* Such scientific validation is imperative to ensure the effectiveness and safety of this traditional Chinese medicine, thereby optimising its medicinal utility.

## Abbreviations

DCM: Dichloromethane
PE: Petroleum ether
